# FAMIN Is a Multifunctional Purine Enzyme Enabling the Purine Nucleotide Cycle

**DOI:** 10.1016/j.cell.2019.12.017

**Published:** 2020-01-23

**Authors:** M. Zaeem Cader, Rodrigo Pereira de Almeida Rodrigues, James A. West, Gavin W. Sewell, Muhammad N. Md-Ibrahim, Stephanie Reikine, Giuseppe Sirago, Lukas W. Unger, Ana Belén Inglesias-Romero, Katharina Ramshorn, Lea-Maxie Haag, Svetlana Saveljeva, Jana-Fabienne Ebel, Philip Rosenstiel, Nicole C. Kaneider, James C. Lee, Trevor D. Lawley, Allan Bradley, Gordon Dougan, Yorgo Modis, Julian L. Griffin, Arthur Kaser

**Affiliations:** 1 Cambridge Institute of Therapeutic Immunology and Infectious Disease, Jeffrey Cheah Biomedical Centre, University of Cambridge, Cambridge CB2 0AW, UK; 2 Division of Gastroenterology and Hepatology, Department of Medicine, University of Cambridge, Addenbrooke’s Hospital, Cambridge CB2 0QQ, UK; 3 Department of Biochemistry and Cambridge Systems Biology Centre, University of Cambridge, Cambridge CB2 1GA, UK; 4 Molecular Immunity Unit, Department of Medicine, University of Cambridge, MRC Laboratory of Molecular Biology, Cambridge CB2 0QH, UK; 5 Institute of Clinical Molecular Biology, Christian Albrechts University, Campus Kiel, 24105 Kiel, Germany; 6 Wellcome Trust Sanger Institute, Hinxton CB10 1SA, UK; 7 Division of Infectious Diseases, Department of Medicine, University of Cambridge, Cambridge CB2 0QQ, UK

**Keywords:** FAMIN, C13orf31, LACC1, purine metabolism, immunometabolism, purine nucleotide cycle, pH homeostasis, redox homeostasis, Crohn's disease, Still's disease

## Abstract

Mutations in FAMIN cause arthritis and inflammatory bowel disease in early childhood, and a common genetic variant increases the risk for Crohn's disease and leprosy. We developed an unbiased liquid chromatography-mass spectrometry screen for enzymatic activity of this orphan protein. We report that FAMIN phosphorolytically cleaves adenosine into adenine and ribose-1-phosphate. Such activity was considered absent from eukaryotic metabolism. FAMIN and its prokaryotic orthologs additionally have adenosine deaminase, purine nucleoside phosphorylase, and *S*-methyl-5′-thioadenosine phosphorylase activity, hence, combine activities of the namesake enzymes of central purine metabolism. FAMIN enables in macrophages a purine nucleotide cycle (PNC) between adenosine and inosine monophosphate and adenylosuccinate, which consumes aspartate and releases fumarate in a manner involving fatty acid oxidation and ATP-citrate lyase activity. This macrophage PNC synchronizes mitochondrial activity with glycolysis by balancing electron transfer to mitochondria, thereby supporting glycolytic activity and promoting oxidative phosphorylation and mitochondrial H^+^ and phosphate recycling.

## Highlights

• An unbiased LC-MS screen reveals FAMIN as a purine nucleoside enzyme
• FAMIN combines adenosine phosphorylase with ADA-, PNP-, and MTAP-like activities
• FAMIN enables a purine nucleotide cycle (PNC) preventing cytoplasmic acidification
• The FAMIN-dependent PNC balances the glycolysis-mitochondrial redox interface

## Graphical Abstract

**Figure:**
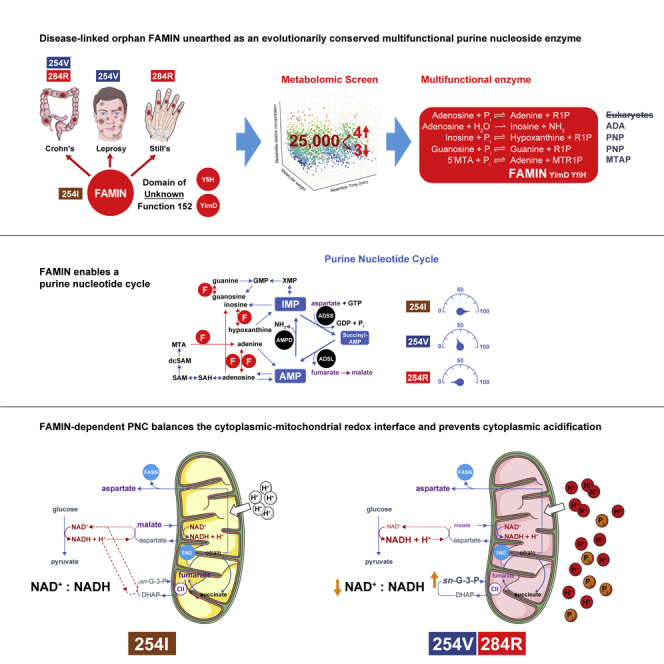

## Teaser

Disease-linked, orphan FAMIN is an evolutionarily conserved, multifunctional purine nucleoside enzyme, with not only ADA-, PNP-, and MTAP-like activities but also adenosine phosphorylase activity. FAMIN enables a purine nucleotide cycle that balances the cytoplasmic-mitochondrial redox interface and prevents cytoplasmic acidification.

## Introduction

FAMIN (Fatty Acid Metabolism-Immunity Nexus; *LACC1*, C13orf31) is strongly linked to human disease (Table S1). Highly penetrant mutations, such as C284R, cause juvenile idiopathic arthritis (JIA), Still's disease (a fever with rash followed by arthritis), or early-onset inflammatory bowel disease (IBD). A common coding polymorphism (I254V) increases risk for Crohn's disease (CD; an IBD) and leprosy, an infection with *Mycobacterium leprae*.

Mice with germline deletion (*Famin*^–/–^) or engineered to express human non-risk, risk, and monogenic disease FAMIN variants (*Famin*^p.254I^, *Famin*^p.254V^, and *Famin*^p.284R^, respectively) revealed that reduced or absent FAMIN activity increases the severity of experimental sepsis and arthritis (Cader et al., 2016, Skon-Hegg et al., 2019). Mitochondrial and NOX2-dependent reactive oxygen species (ROS) generation, bacterial killing, NOD2- and Toll-like receptor (TLR)-dependent signaling, inflammasome activation, and cytokine secretion are compromised with impaired FAMIN and linked to perturbed mitochondrial function (Cader et al., 2016, Lahiri et al., 2017). Oxidative phosphorylation (OXPHOS) and glycolysis are compromised and total cellular adenosine triphosphate (ATP) reduced in *Famin* mutant macrophages. Impaired FAMIN compromises both classically activated “M1” macrophages and alternatively activated “M2” macrophages (Cader et al., 2016, O’Neill and Pearce, 2016). FAMIN tethers to the cytosolic surface of peroxisomes in a complex with fatty acid synthase (FASN) (Assadi et al., 2016, Cader et al., 2016, Hillebrand et al., 2012). The flux of glucose carbon into fatty acid synthesis and fatty acid oxidation (FAO) is curtailed in *Famin* mutant macrophages (Cader et al., 2016). How FAMIN, which shares homology with bacterial orthologs (Pfam motif Domain of Unknown Function [DUF] 152), exerts such profound immunometabolic control had remained enigmatic.

Here, we report an unbiased metabolomic screen for enzyme activity that unearthed FAMIN as a conserved multi-functional purine nucleoside metabolizing enzyme with activities that challenge fundamental principles of purine metabolism.

## Results

### Unbiased High-Complexity Metabolomic Screen

**Figure S1:**
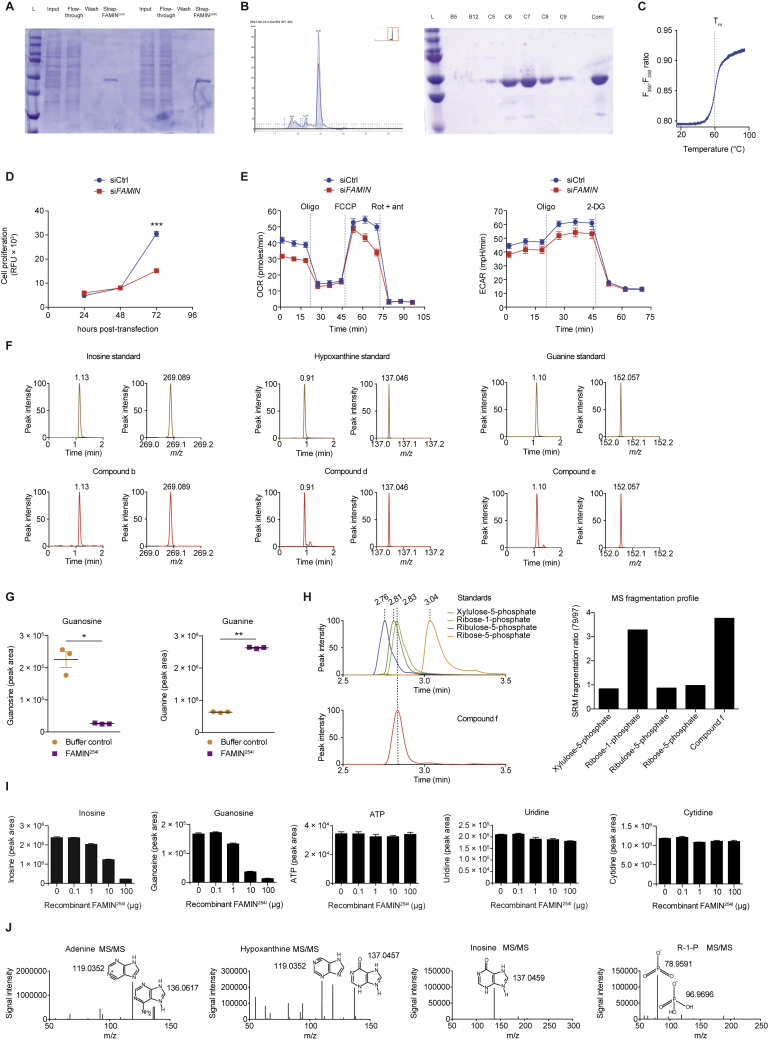
FAMIN Metabolizes Purine Nucleosides, Related to Figure 1 (A) Coomassie SDS-PAGE of recombinant human FAMIN^254I^ and FAMIN^254V^ following Strep-Tactin affinity purification. Lanes indicate ladder (L), FAMIN^254I^ or FAMIN^254V^ transfected HEK293T lysate input, column flow-through and concentrated protein eluate. (B) Left, size exclusion chromatogram of affinity purified FAMIN that has undergone TEV-cleavage to remove Strep-tag. Blue trace corresponds to A280 (protein) and purple trace to A260 (DNA) signal. Fractions C6-C8 were collected, concentrated, and subjected to Coomassie SDS-PAGE. Inset depicts entire chromatogram. Right, Coomassie SDS-PAGE of fractions obtained from size exclusion chromatography. Lanes indicate ladder (L) and fractions B12, C5, C6, C7, C8 and C9, corresponding to the size exclusion chromatogram, and the concentrated protein from fractions C6-C8. (C) Differential scanning fluorimetry (DSF) of recombinant human FAMIN. (D) Cell proliferation of HepG2 cells silenced for FAMIN (si*FAMIN*) or transfected with scrambled siRNA (siCtrl) as measured by CyQUANT assay (n = 12). (E) Oxygen consumption rate (OCR) and extracellular acidification rate (ECAR) of HepG2 cells 48 h after transfection with *FAMIN* or control siRNA. Basal OCR measurement was followed by sequential treatment (dotted vertical lines) with oligomycin A (Oligo), FCCP, and rotenone plus antimycin A (Rot + ant). Basal ECAR measurement was followed by sequential treatment with oligomycin (Oligo) and 2-deoxyglucose (2-DG) (n = 3). (F) Representative mass spectra and extracted chromatograms for putative FAMIN-catalyzed metabolites and corresponding standards for inosine, hypoxanthine and guanine. (G) Guanosine and guanine levels following incubation of HepG2 cell aqueous extract with 10 μg recombinant FAMIN^254I^ in 100 μL PBS. (n = 3). (H) Left, Representative extracted chromatograms for FAMIN-catalyzed compound ‘f’ and corresponding standards for ribose-1-phosphate, ribose-5-phosphate, ribulose-5-phosphate and xylulose-5-phosphate. All measurements performed using a BEH amide HILIC column and TSQ Quantiva triple quadrupole. Right, Ratio of selected reaction monitoring (SRM) daughter ions with nominal *m/z* values of 79 and 97. (I) Inosine, guanosine, cytidine, uridine and ATP levels following incubation of 0.1, 1.0, 10.0 or 100.0 μg of recombinant FAMIN^254I^ with the complete metabolomic library (aqueous phase of methanol:chloroform extract of *FAMIN*-silenced HepG2 cells) in 100 μL PBS (n = 3). (J) LC-MS peaks putatively identified as adenine, hypoxanthine, inosine, or ribose-1-phosphate with nominal *m/z* values of 136, 137, 269 and 229, respectively, were selectively targeted and fragmented using a higher-energy collision dissociation (HCD) collision voltage of 25 eV to give the fragments shown. Data are represented as mean ± SEM or representative of at least 3 independent experiments. ^∗^p < 0.05, ^∗∗^p < 0.01, ^∗∗∗^p < 0.001 (unpaired, two-tailed Student’s t test).

Identifying biochemical functions of orphan proteins is a formidable challenge (Prosser et al., 2014). We devised an unbiased screen for enzyme activity against an extensive library of metabolites without *a priori* assumptions on putative function. From transiently transfected HEK293T cells, we purified chromatographically monomeric recombinant human FAMIN (referred to as “FAMIN^254I^” for the fully active variant), which exhibited stable properties in solution consistent with correct folding and lack of aggregation (Figures S1A–S1C). We generated a metabolite library from the human hepatocellular carcinoma cell line HepG2 transfected with *FAMIN* small interfering RNA (siRNA), which proliferated less and exhibited reduced glycolysis and OXPHOS (Figures S1D and S1E). Hence, FAMIN performed a non-redundant role, letting us expect that extracts would contain all cofactors and substrates required for its activity.

**Figure 1:**
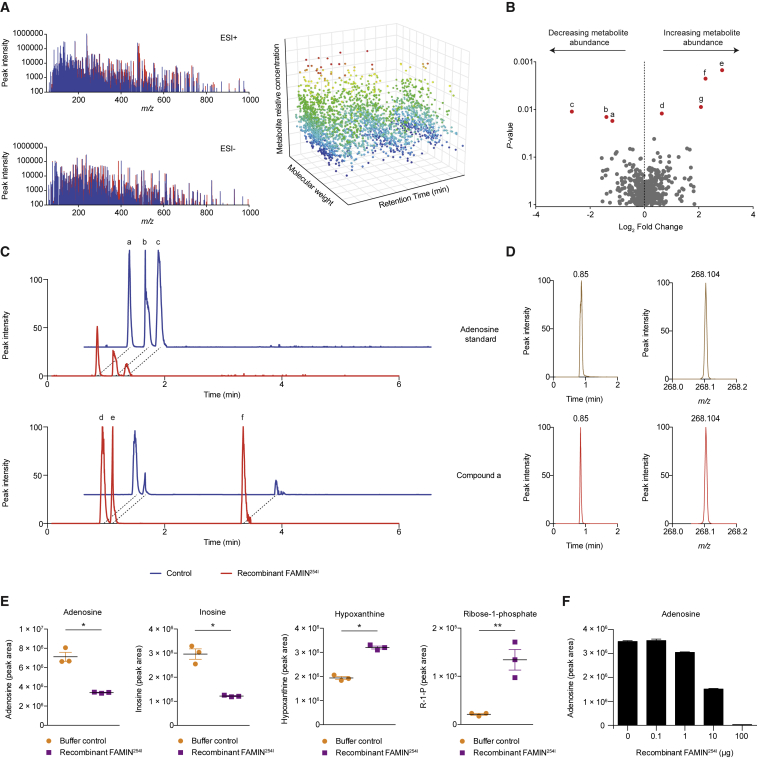
FAMIN Is a Purine-Nucleoside-Metabolizing Enzyme (A) Metabolomic library of HepG2 cells after transfection with *FAMIN* siRNA. Representative total mass spectra (left) separated by molecular weight (*m/z*), chromatography retention time, and relative levels (right). (B) Change in relative metabolite levels in the library after incubation with recombinant FAMIN^254I^ or protein buffer control, depicted as volcano plot with unadjusted p values. Red dots, candidate substrates and products whose abundance decreased (a–c) or increased (d–f; n = 3 independent reactions). (C) Representative extracted chromatograms for candidate substrates (top) and products (bottom) by using normalized peak intensity for each given *m/z* value. (D) Representative mass spectra and extracted chromatograms for compound a and corresponding authentic standard. (E) Levels of adenosine, inosine, hypoxanthine, and ribose-1-phosphate (R1P) within the metabolomic library incubated with FAMIN^254I^ or protein buffer control (n = 3, mean ± SEM). (F) Levels of adenosine within the metabolomic library incubated with 0.1–100 μg of FAMIN^254I^ or protein buffer control (n = 3, mean ± SEM). Data representative of at least 3 independent experiments. ^∗^p < 0.05 and ^∗∗^p < 0.01 (unpaired, two-tailed Student’s t test).

We adopted quantitative, high-sensitivity and high-resolution orthogonal liquid chromatography-mass spectrometry (LC-MS) to resolve a wide range of highly diverse metabolites. We identified >25,000 unique quantifiable LC-MS features in freeze-dried aqueous extracts of *FAMIN*-silenced HepG2 cells across chromatography modalities and ionization modes (Figure 1A). We incubated 10 μg recombinant human FAMIN^254I^ or an equivalent volume of protein buffer, with the metabolite library resuspended in phosphate-buffered saline (PBS) (pH 7.4) for 1 h at 37°C. Samples were then re-extracted and analyzed. Within this vast library, 3 LC-MS features significantly decreased and 4 increased in abundance in the FAMIN^254I^ compared to the mock reaction (Figures 1B and 1C).

The *m/z* values of the 3 LC-MS features with reduced abundance (a–c in Figures 1B and 1C) matched exactly those of purine nucleosides. Molecular formula determination, using accurate mass and supported by isotopic mass distribution, also indicated compounds a–c were purine nucleosides. Molecular formula and *m/z* could not unambiguously discriminate their identity. Comparing chromatography characteristics of a–c against authentic standards demonstrated that the retention times exactly matched adenosine, inosine, and guanosine (Figures 1D, 1E, S1F, and S1G), suggesting FAMIN catabolizes the major cellular purine nucleosides. Consistent with this, the *m/z* values of LC-MS features d–f (Figures 1B and 1C), whose levels increased in the presence of FAMIN^254I^, matched hypoxanthine, guanine, and a pentose-phosphate, respectively (Figures 1E and S1G). LC-MS feature g corresponded to xanthine, although levels were extremely low. Modified chromatography unambiguously separated isomeric pentose-phosphates and identified feature f as ribose-1-phosphate (R1P; Figure S1H). Recombinant FAMIN^254I^ did not affect any other nucleosides or nucleotides also present in our library (Figure S1I). Adenosine, inosine, and guanosine consumption increased with the amount of recombinant FAMIN^254I^ in the reaction (Figures 1F and S1I). This suggested FAMIN may be an enzyme acting on purine nucleosides to generate nucleobases and R1P.

### FAMIN Combines Adenosine Deaminase, Purine Nucleoside Phosphorylase, and *S*-Methyl-5′-Thioadenosine Phosphorylase Activities

**Figure 2:**
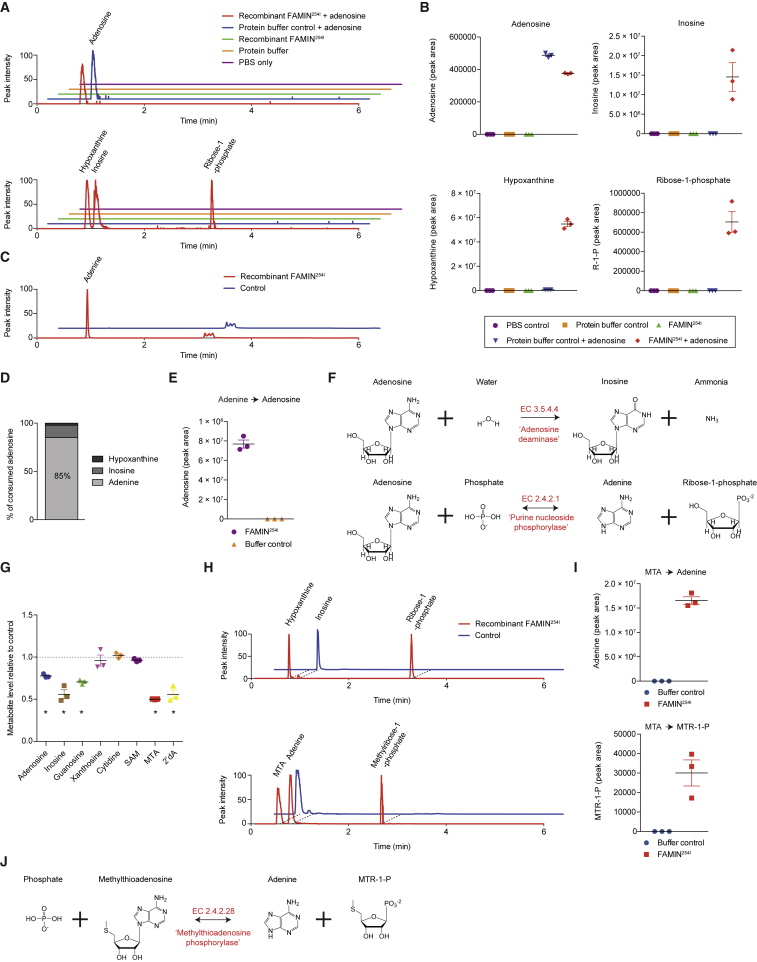
FAMIN Has Adenosine Deaminase, Purine Nucleoside Phosphorylase, and *S*-Methyl-5′-Thioadenosine (MTA) Phosphorylase Activities (A) Representative extracted chromatograms, using normalized peak intensity, for adenosine (top chromatogram); and inosine, hypoxanthine, and R1P (bottom chromatogram) following incubation of FAMIN^254I^ or control with 100 μM adenosine. (B) Adenosine, inosine, hypoxanthine, and R1P levels following incubation of recombinant FAMIN^254I^ or control as per (A) (n = 3). (C) Representative extracted chromatograms for adenine using a modified CSH-C18 method. (D) Fractional conversion of adenosine into its products following incubation of Strep-tagged FAMIN^254I^ with 100 μM adenosine (n = 3, mean). (E) Adenosine levels in reactions of adenine and R1P in the presence of Strep-tagged FAMIN^254I^ or control (n = 3). (F) FAMIN-catalyzed enzymatic reactions. (G) FAMIN activity toward purine and pyrimidine nucleosides, measured as substrate (each added at 100 μM) consumption (SAM; *S*-adenosylmethionine; 2′-dA, 2′-deoxyadenosine; n = 3). (H) Representative extracted chromatograms for inosine, hypoxanthine, and R1P (top chromatogram); and MTA, adenine, and methylthioribose-1-phosphate (bottom chromatogram) upon incubation of inosine and MTA, respectively, with recombinant FAMIN^254I^. (I) Adenine and methylthioribose-1-phosphate levels upon incubation of MTA with FAMIN^254I^ or buffer control (n = 3). (J) Further FAMIN-catalyzed enzymatic reaction. Data represented as mean ± SEM. ^∗^p < 0.05 (unpaired, two-tailed Student’s t test).

**Figure S2:**
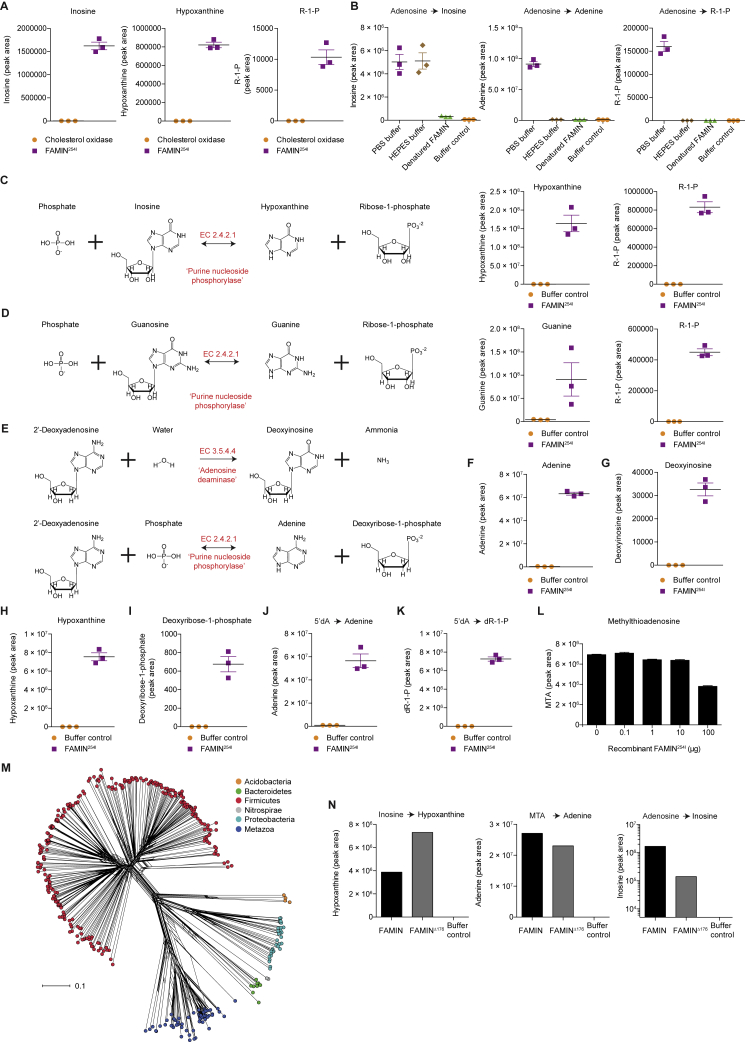
Characterization of FAMIN Enzymatic Activity, Related to Figure 2 (A) Inosine, hypoxanthine and ribose-1-phosphate levels following incubation of 10 μg recombinant FAMIN^254I^ or equimolar cholesterol oxidase with 10 μM adenosine for 1 h in 100 μL PBS (n = 3). (B) Adenine, inosine and ribose-1-phosphate levels following incubation of 10 μg recombinant Strep-tagged FAMIN^254I^ or appropriate controls, including heat-denatured recombinant Strep-tagged FAMIN^254I^, with 10 μM adenosine for 1 h in 100 μL PBS or HEPES buffer (n = 3). (C and D) Left, FAMIN-catalyzed enzymatic reactions. Right, levels of guanine or hypoxanthine and ribose-1-phosphate in reactions containing 100 μM guanosine or inosine and recombinant FAMIN^254I^ or buffer control in 100 μL after 1 h at 37°C (n = 3). (E–I) FAMIN-catalyzed enzymatic reaction with (F) adenine, (G) 2′-deoxyinosine, (H) hypoxanthine and (I) deoxyribose-1-phosphate levels following incubation of 10 μg recombinant Strep-tagged FAMIN^254I^ or buffer control with 10 μM 2′deoxyadenosine for 1 h in 100 μL PBS (n = 3). (J and K) Adenine (J) and deoxyribose-1-phosphate (K) levels following incubation of 10 μg recombinant Strep-tagged FAMIN^254I^ or buffer control with 10 μM 5′deoxyadenosine (5′dA) for 1 h in 100 μl PBS (n = 3). (L) MTA levels following incubation of 0.1, 1.0, 10.0 or 100.0 μg of recombinant FAMIN^254I^ with the complete metabolomic library (aqueous phase of methanol:chloroform extract of *FAMIN*-silenced HepG2 cells) in 100 μl PBS (n = 3). (M) Phylogenetic tree of FAMIN orthologs using human FAMIN protein sequence as the search input. (N) EC 3.5.4.4 (Adenosine deaminase), EC 2.4.2.1 (purine nucleoside phosphorylase) and EC 2.4.2.28 (MTA phosphorylase) activities of *E. coli* expressed recombinant full-length FAMIN and FAMIN^Δ176^ as measured by inosine, hypoxanthine and adenine production following incubation of protein with 10 μM adenosine, inosine and MTA, respectively, in PBS. Data are represented as mean ± SEM. ^∗^p < 0.05, ^∗∗^p < 0.01, ^∗∗∗^p < 0.001 (unpaired, two-tailed Student’s t test).

To unambiguously validate results from the library screen, we examined enzyme activity in a fully reductionist system using pure substrate. FAMIN^254I^ consumed adenosine and generated inosine, hypoxanthine, and R1P, which was confirmed by authentic standards (Figures 2A, 2B, and S1J). No spontaneous degradation of adenosine or formation of products occurred in the absence of FAMIN^254I^ or adenosine (Figures 2A and 2B), nor with an unrelated enzyme, cholesterol oxidase (Figure S2A). Because our LC methods did not resolve adenosine and adenine well and because adenosine can undergo source fragmentation to adenine, we applied further chromatography methods to separate them. This demonstrated that FAMIN^254I^ converted adenosine to adenine (Figure 2C), whereby ∼85% of consumed adenosine yielded adenine and ∼15% inosine (Figure 2D). FAMIN-catalyzed activities were further confirmed by tracing [^15^N_5_^13^C_10_] adenosine-derived stable isotopes into reaction products (Table S2). Incubating FAMIN^254I^ with adenine and R1P yielded adenosine, demonstrating the reverse reaction (Figure 2E) and corroborating identities of the products of the forward reaction. No other adenosine products were detected, and heat-denaturing inactivated FAMIN^254I^ (Figure S2B). Consistent with orthophosphate (P_i_) required for phosphorolysis, the reaction progressed only to inosine when performed in non-phosphate buffer (Figure S2B). Hence, FAMIN exhibited activities as adenosine deaminase and purine nucleoside phosphorylase, and reactions proceeded independently from each other (Figure 2F).

FAMIN^254I^ also metabolized pure inosine and guanosine into R1P and their respective nucleobases hypoxanthine and guanine (Figures 2G, 2H, S2C, and S2D), whereas no activity toward cytidine was detected (Figure 2G). FAMIN^254I^ also metabolized 2′-deoxy-adenosine, producing adenine, 2′-deoxy-inosine, hypoxanthine, and 2′-deoxy-R1P (Figures 2G and S2E–S2I). 5′-Deoxy-adenosine, a by-product of radical *S*-adenosylmethionine (SAM) enzymes (Landgraf et al., 2016), was also a substrate of FAMIN^254I^ (Figure S2J and S2K). FAMIN^254I^ further metabolized *S-*methyl-5′-thioadenosine (MTA) into adenine and *S*-methyl-5′-thioribose-1-phosphate (Figures 2H and 2I), while not affecting SAM (Figure 2G). Revisiting the library screen, we noticed a 50% reduction of MTA with 100 μg FAMIN^254I^ (Figure S2L). This added a third activity of FAMIN as a MTA phosphorylase (Figure 2J).

### FAMIN’s Purine Nucleoside Enzymatic Activities Are Conserved

**Figure 3:**
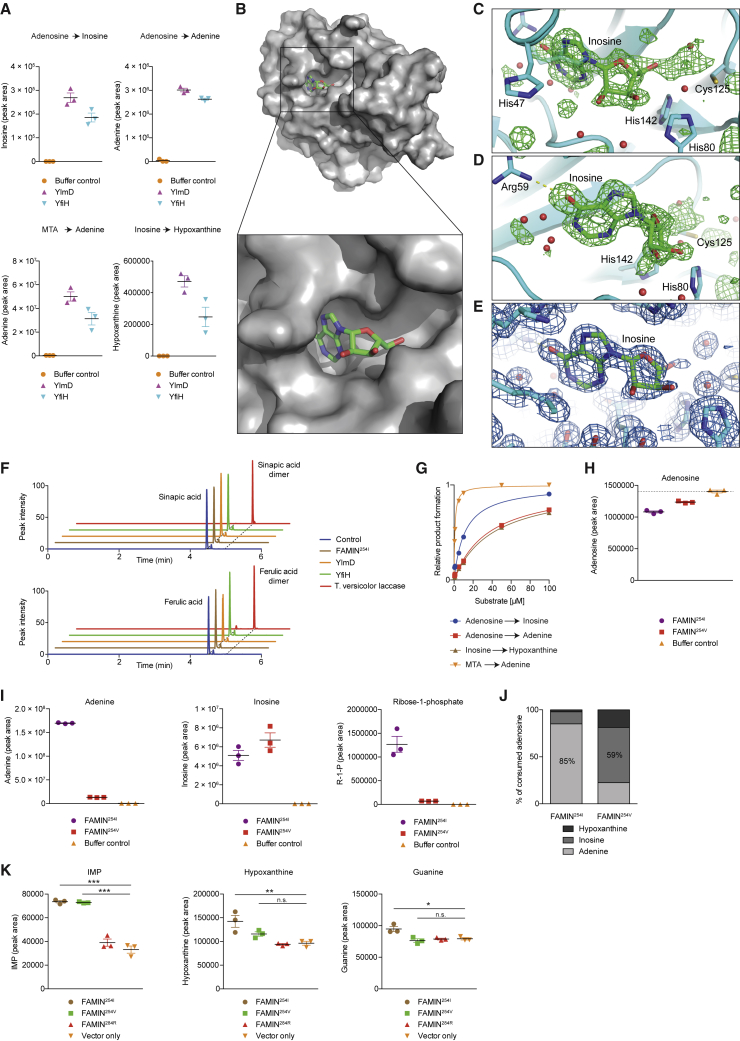
FAMIN Activities Are Evolutionarily Conserved and Adenosine Phosphorolysis Compromised in I254V (A) Enzyme activities of YlmD and YfiH as measured by inosine, adenine, and hypoxanthine production (n = 3). (B) Crystal structure of YlmD determined in the presence of inosine and phosphate, shown in molecular surface representation with a bound inosine as ball-and-stick. (C and D) Substrate binding site of YlmD, polder *Fo-Fc* electron density map calculated at 1.5-Å resolution with inosine and bulk solvent omitted. Maps contoured at +3.5 σ (green mesh). (C) Cys^125^-His^80^-His^142^ located near inosine’s ribose moiety. The His^47^ side chain inserted into the purine-binding pocket in apo-YlmD (semi-transparent representation). (D) View rotated 45° around the y axis. The hypoxanthine moiety forms a hydrogen bond with the Arg^59^ side chain (dashed line). Selected ordered water molecules (red spheres). (E) 2*Fo-Fc* electron density map near the bound inosine calculated after refinement with diffraction data to 1.2-Å resolution and contoured at +1.2 σ (blue mesh). Viewing orientation between those in (C) and (D). (F) Representative extracted chromatograms demonstrating oxidation of laccase substrates sinapic and ferulic acid into dimer products after incubation with YlmD, YfiH, Strep-tagged FAMIN^254I^, laccase from *Trametes versicolor*, or appropriate control. (G) Michaelis-Menten kinetics of FAMIN activities for indicated substrates. (H and I) Consumption of adenosine (H) and production of adenine, inosine, and R1P (I) following incubation of Strep-tagged FAMIN^254I^, FAMIN^254V^, or buffer control with 100 μM adenosine (n = 3). (J) Fractional conversion of adenosine into adenine versus inosine versus hypoxanthine following incubation of adenosine with Strep-tagged FAMIN^254I^ or FAMIN^254V^ (n = 3, mean). (K) Inosine monophosphate (IMP), hypoxanthine, and guanine levels in HEK293T cells after transient transfection with FAMIN expression vectors or empty vector (n = 3). Data represented as mean ± SEM. ^∗^p < 0.05, ^∗∗^p < 0.01, and ^∗∗∗^p < 0.001 (unpaired, two-tailed Student’s t test).

**Figure S3:**
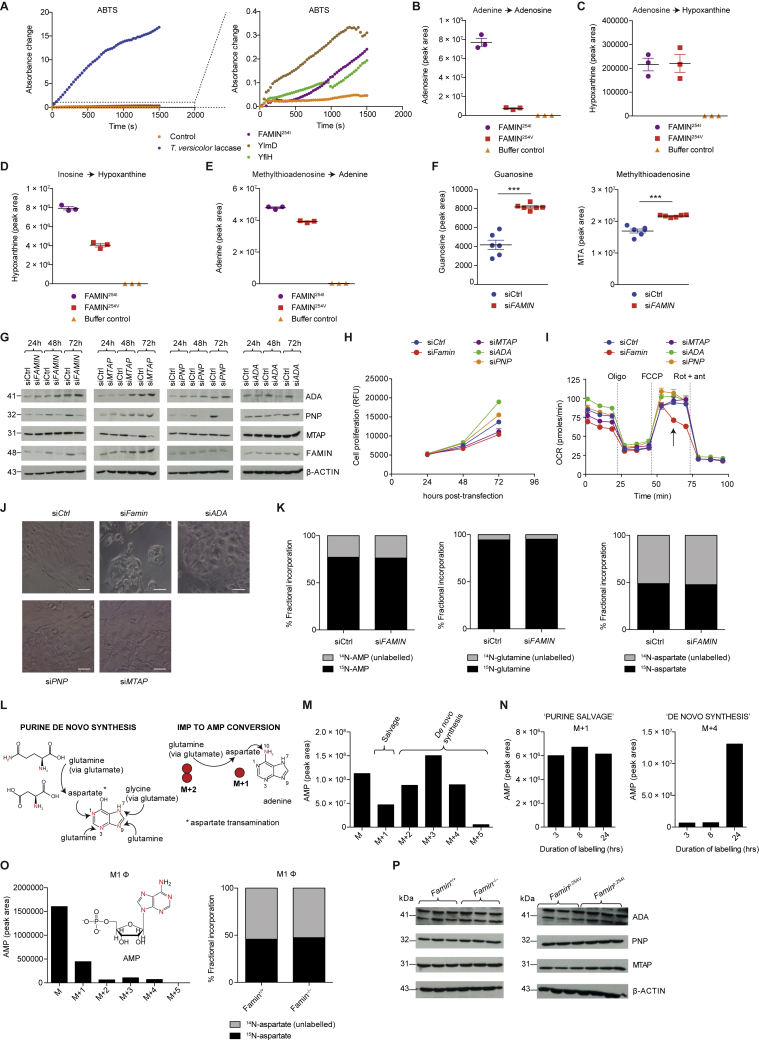
FAMIN Enzymatic Activity Determines Cellular Purine Metabolism and Is Impaired in FAMIN^254V^, Related to Figure 3 (A) Laccase enzyme activity of recombinant YlmD, YfiH, FAMIN^254I^ and *Trametes versicolor* laccase using 2,2′-azino-bis-3-ethylbenzothiazoline-6-sulphonic acid (ABTS). Please note the right panel’s y axis, which shows a graphical enlargement of the low absorbance readings from the left panel. Data are representative of 2-3 independent experiments. (B) Adenosine levels following incubation of 10 μg Strep-tagged FAMIN^254I^, FAMIN^254V^ or control with 50 μM adenine and 50 μM ribose-1-phosphate for 1 h in 100 μL PBS. (C) Hypoxanthine levels following incubation of 10 μg recombinant Strep-tagged FAMIN^254I^ or FAMIN^254V^ with 10 μM adenosine for 1 h in 100 μL phosphate buffered saline (PBS), (n = 3). (D) EC 2.4.2.1 (purine nucleoside phosphorylase) and (E) EC 2.4.2.28 (MTA phosphorylase) activities of Strep-tagged FAMIN^254I^ and FAMIN^254V^ as measured by hypoxanthine and adenine following incubation of recombinant protein with 10 μM inosine and methylthioadenosine (MTA), respectively, in PBS (n = 3). (F) Guanosine and *S*-methyl-5′-thioadenosine levels in control and *FAMIN*-silenced HepG2 cells 48 h after transfection (n = 6). (G) Immunoblots (IB) for ADA, PNP, MTAP and FAMIN from HepG2 cell lysates silenced for FAMIN (si*FAMIN*), adenosine deaminase (si*ADA*), purine nucleoside phosphorylase (si*PNP*), methylthioadenosine phosphorylase (si*MTAP*) or scrambled siRNA (siCtrl) at 24 h, 48 h or 72 h following transfection; β-ACTIN loading control. (H) Cell proliferation over time of control, *FAMIN*, *ADA*, *PNP* or *MTAP* silenced HepG2 cells as measured by CyQUANT assay (n = 12). (I) Oxygen consumption rate (OCR) of HepG2 cells transfected with si*FAMIN*, si*ADA*, si*PNP*, si*MTAP* or siCtrl. Basal OCR measurement was followed by sequential treatment (dotted vertical lines) with oligomycin A (Oligo), FCCP, and rotenone plus antimycin A (Rot + ant), (n = 3). Arrow indicates steep decline observed in siFAMIN cells after treatment with FCCP. (J) Cell morphology by light microscopy of HepG2 cells silenced with si*FAMIN*, si*ADA*, si*PNP*, si*MTAP* or si*Ctrl* as observed at 72 h after transfection; scale bar = 125 μm. Data are representative of at least 3 independent experiments. (K–O) A 24h pulse with ^15^N_2_-glutamine labeled three quarters of AMP in HepG2 cells (n = 3; mean). Since this pulse labeled almost all cellular glutamine and ∼50% of aspartate, the number of incorporated ^15^N atoms into AMP allowed (L–M) estimating the proportion of purine *de novo* synthesis (M+2, M+3, M+4 isotopomers) versus salvage via HPRT (M+1 isotopomer). (N) HepG2 cells engaged both purine salvage and *de novo* synthesis, but as expected with different kinetics. (O) Terminally differentiated M1Φ, in contrast, exhibited very little *de novo* synthesis, Levels of M, M+1, M+2, M+3, M+4, M+5 labeled forms of AMP in M1 macrophages after a 24 h pulse with ^15^N_2_-glutamine (n = 3; mean). The M+1 isotopomer might substantially underestimate salvage, since only half of aspartate is labeled and any HPRT-dependent salvage subsequent to *de novo* synthesis, or salvage via APRT, would not be captured. This high turnover of the purine ring extends to all essential metabolites and cofactors that contain adenyl groups, i.e., coenzyme A (CoA), acetyl-CoA, flavin adenine dinucleotide (FAD), nicotinamide adenine dinucleotide (NAD), NAD phosphate (NADP), SAM, SAH and MTA (data not shown). (P) Immunoblots (IB) for adenosine deaminase (ADA), purine nucleoside phosphorylase (PNP) and *S*-methyl-5′-thioadenosine phosphorylase (MTAP) in *Famin*^+/+^ and *Famin*^–/–^ and *Famin*^p.254V^ and *Famin*^p.254I^ M1 macrophages; β-actin, loading control. Data are represented as mean ± SEM. ^∗^p < 0.05, ^∗∗^p < 0.01, ^∗∗∗^p < 0.001 (unpaired, two-tailed Student’s t test).

Orthologs containing the C-terminal portion of FAMIN are widely distributed across prokaryotes but are confined to metazoans in eukaryotes (Figure S2M). An *Escherichia coli*-expressed, maltose-binding protein (MBP) fusion protein of truncated FAMIN containing the DUF152 homology domain only (FAMIN^Δ176^) exhibited all three enzymatic activities, similar to MBP-tagged full-length FAMIN (Figure S2N). Consistent with DUF152 containing all enzymatic activity, recombinantly expressed DUF152 bacterial proteins YlmD and YfiH metabolized adenosine to inosine and adenine, MTA to adenine, and inosine to hypoxanthine (Figure 3A). Crystal structures of several bacterial DUF152 proteins have been determined and revealed a monomeric α/β/α fold (Kim et al., 2006). To identify the binding pocket, we soaked YlmD crystals with inosine. Inosine-soaked crystals diffracted to 1.2-Å resolution and contained additional electron density not present in native YlmD crystals, unambiguously identifying the purine ring of a bound inosine molecule (Figure 3B; Table S3). The structure contained weaker additional density of a partially hydrolyzed or disordered ribose moiety (Figures 3C–3E). The hypoxanthine moiety forms a hydrogen bond with the side chain of Arg^59^. The ribose moiety is coordinated by the side chains of Cys^125^, His^80^, and His^142^, a triad conserved in YfiH and previously predicted as active site (Kim et al., 2006). In contrast to inosine-bound YlmD, in an apo-YlmD crystal that diffracted to 1.2 Å, the His^47^ side chain was found inserted into the purine-binding pocket (Figure 3C), consistent with a previously deposited apo structure (protein data bank [PDB]: 1T8H). The His^47^ side chain may act as a gatekeeper of the active site that rotates outward when the substrate binds. FAMIN has several aromatic residues in the corresponding loop that could function analogously to His^47^. Atypical laccase activity has been ascribed to some bacterial DUF152 proteins based on a spectrophotometric method that monitors the oxidation of proxy substrates (Beloqui et al., 2006). In this method, YlmD, YfiH, and FAMIN^254I^, in contrast to a conventional laccase, elicited only minuscule signals (Figure S3A). In a sensitive LC-MS method (Perna et al., 2018), laccase end-products were only detected with a conventional laccase but not with YlmD, YfiH, or FAMIN^254I^ (Figure 3F). We conclude that FAMIN is a prototype of a new, evolutionarily conserved family of multifunctional purine-nucleoside-metabolizing enzymes.

### FAMIN Combines Adenosine Phosphorylase with ADA-, PNP-, and MTAP-like Activities

Eukaryotic cells have been considered devoid of an enzyme that phosphorolytically converts adenosine to adenine (Friedkin and Kalckar, 1961, Maynes et al., 1999, Zimmerman et al., 1971). FAMIN adds such adenosine phosphorylase activity to mammalian metabolism and combines within one single enzyme three essential, non-redundant activities that supply purine nucleotide salvage. These latter activities had been thought to be due to single, ubiquitously expressed genes: adenosine deaminase (*ADA*; secreted ADA2 is expressed from a separate gene), purine nucleoside phosphorylase (*PNP*), and *S*-methyl-5′-thioadenosine phosphorylase (*MTAP*) (Ashihara et al., 2018, Murray, 1971). Loss of ADA or PNP activity causes severe combined immunodeficiency (Giblett et al., 1972, Giblett et al., 1975), whereas *MTAP* is frequently deleted in cancers (Kryukov et al., 2016). ADA, PNP, and MTAP activities are critically important because adenosine, inosine, and guanosine and their nucleobases adenine, hypoxanthine, and guanine are neither precursors nor intermediates of *de novo* purine synthesis but are generated by the reactions that supply purine nucleotide salvage; salvage then proceeds by hypoxanthine guanine phosphoribosyl transferase (HPRT) and adenine phosphoribosyl transferase (APRT) (Camici et al., 2018).

### I254V Switches Activity from Adenosine Phosphorolysis to Deamination

FAMIN’s apparent *K*_m_s for its main substrates were similar to those reported for ADA, PNP, and MTAP (Figure 3G; Table S4; Bzowska et al., 2000, Della Ragione et al., 1996, Lindley and Pisoni, 1993), although the corresponding *V*_max_s were low (Table S4). We next addressed how I254V affected activity. Adenosine consumption was approximately halved with FAMIN^254V^ compared to FAMIN^254I^ (Figure 3H), with conversion to adenine and R1P 10-fold lower (Figure 3I), mirrored in the reverse reaction (Figure S3B). In contrast, the production of inosine and hypoxanthine by FAMIN^254V^ was slightly higher and similar, respectively, compared to FAMIN^254I^ (Figures 3I and S3C). Thus, FAMIN^254V^ converted only ∼20%, rather than FAMIN^254I^’s 85%, of consumed adenosine to adenine, instead diverting it to inosine and thence hypoxanthine (Figure 3J). Phosphorolysis activities toward inosine and MTA were also lower in FAMIN^254V^ than in FAMIN^254I^ (Figures S3D and S3E). Hence, FAMIN^254I^ and FAMIN^254V^ activities differed quantitatively and qualitatively, favoring adenosine phosphorolysis and adenosine deamination, respectively.

### FAMIN Controls Cellular Purine Levels

**Figure 4:**
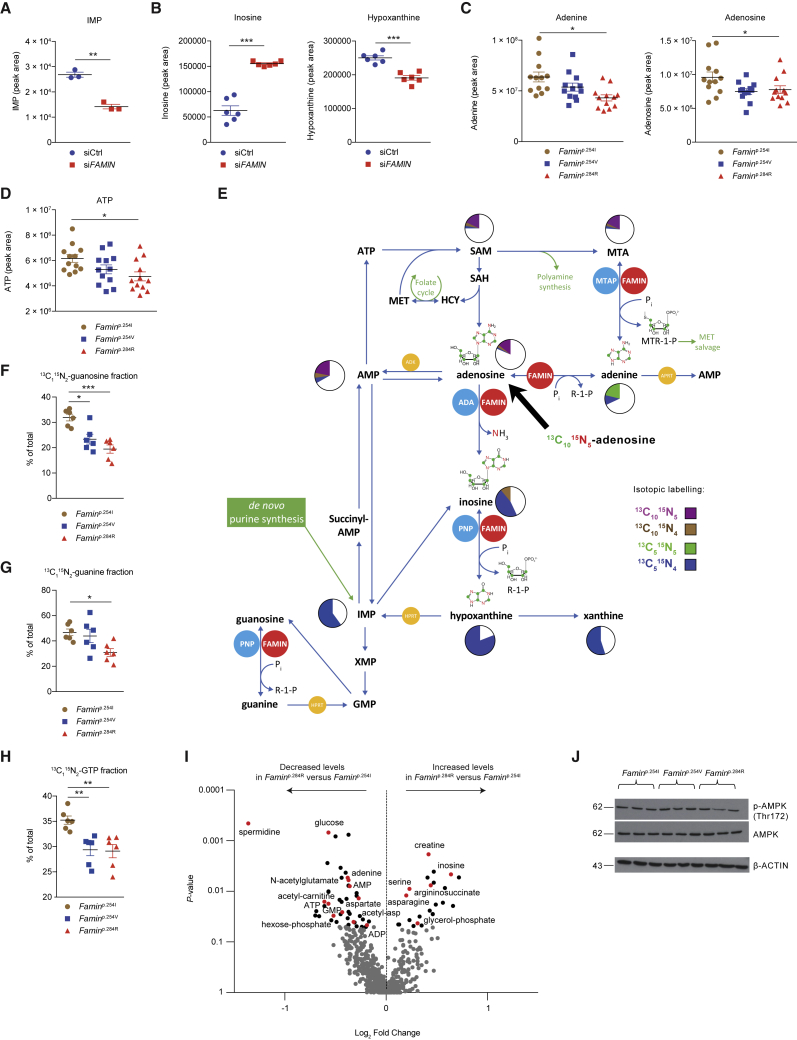
FAMIN Variants Impact on Central Purine Routing (A) IMP levels in control and *FAMIN*-silenced HepG2 cells 24 h after transfection (n = 3). (B) Inosine and hypoxanthine levels in control and *FAMIN*-silenced HepG2 cells 48 h after transfection (n = 6). (C and D) Adenine, adenosine, (C) and ATP levels (D) in *Famin*^p.254I^, *Famin*^p.254V^, and *Famin*^p.284R^ M1 macrophages (n = 12). (E) Metabolic fate of [^13^C_10_^15^N_5_] adenosine after a 3-h pulse of M1 macrophages (n = 6; mean). Schematic representation of central purine metabolism. Adenosine deamination into inosine releases ^15^N as ammonia, generating a [^13^C_10_^15^N_4_] isotopomer (brown). Phosphorolytic cleavage of inosine into hypoxanthine and [^13^C_5_] R1P, yielding the [^13^C_5_^15^N_4_] isotopomer (blue). Adenosine conversion to AMP without loss of label (purple). Phosphorolytic cleavage of fully labeled MTA generates [^13^C_5_^15^N_5_] adenine (green) and [^13^C_5_] 5′-methylthioribose-1-phosphate. Fractions of differently labeled states (averaged across *Famin*^p.254I^, *Famin*^p.254V^, and *Famin*^p.284R^ genotypes) depicted as pie charts. ADA, adenosine deaminase; ADK, adenosine kinase; APRT, adenine phosphoribosyl transferase; HPRT, hypoxanthine-guanine phosphoribosyl transferase; MTAP, MTA phosphorylase; PNP, purine nucleoside phosphorlyase. (F–H) Fraction of guanosine (F), guanine (G), or GTP (H) labeled as the indicated isotopomer in M1 macrophages after a [^13^C_1_^15^N_2_] guanosine pulse (n = 6). (I) Metabolite levels (gray dots) in *Famin*^p.254I^ versus *Famin*^p.284R^ M1 macrophages depicted as volcano plot. False discovery rate (FDR)-controlled LC-MS features (black dots), select metabolites in red (n = 6). (J) Immunoblots (IBs) with indicated antibodies in M1 macrophages (n = 3). Data represented as mean ± SEM. ^∗^p < 0.05, ^∗∗^p < 0.01, and ^∗∗∗^p < 0.001 (unpaired, two-tailed Student’s t test or one-way ANOVA).

To address whether FAMIN affected cellular purine levels, we transiently transfected FAMIN expression vectors into HEK293T cells. FAMIN^254I^ and FAMIN^254V^ proteins express equally high, and FAMIN^284R^ expression is negligible (Cader et al., 2016). Hypoxanthine, guanine, and inosine monophosphate (IMP) levels were higher 24 h after transfecting FAMIN^254I^ than in controls (Figure 3K). The doubling in levels of IMP, the obligatory intermediate of purine salvage and *de novo* synthesis (Camici et al., 2018), extended to FAMIN^254V^ transfectants, whereas the increase in hypoxanthine was moderate compared to FAMIN^254I^ (Figure 3K). Other purine nucleosides were not affected (Table S5). Conversely, IMP levels halved in HepG2 cells 24 h after transfecting *FAMIN* siRNA (Figure 4A), which, compared to control siRNA, boosted inosine, guanosine, and MTA and reduced hypoxanthine levels (Figures 4B and S3F). Hence, FAMIN impacted on the levels of central purines despite preserved ADA, PNP, and MTAP expression (Figure S3G), prompting us to compare proliferation and energy metabolism upon their knockdown. Although *MTAP* siRNA reduced proliferation (Figure S3H) and basal respiration similar to *FAMIN* siRNA, *MTAP*-silenced cells did not exhibit the steep decline in respiration of *FAMIN*-silenced HepG2 cells after eliciting spare respiratory capacity (SRC) (Figure S3I). Changes in cell shape were distinct between *MTAP* and *FAMIN* siRNA transfection (Figure S3J), whereas *ADA* and *PNP* siRNA affected proliferation and respiration overall differently from *FAMIN* siRNA (Figures S3H and S3I). Hence, FAMIN and the monofunctional enzymes did not cross-compensate for their individual absence.

### FAMIN Affects Routing through Core Purine Metabolism

**Figure S4:**
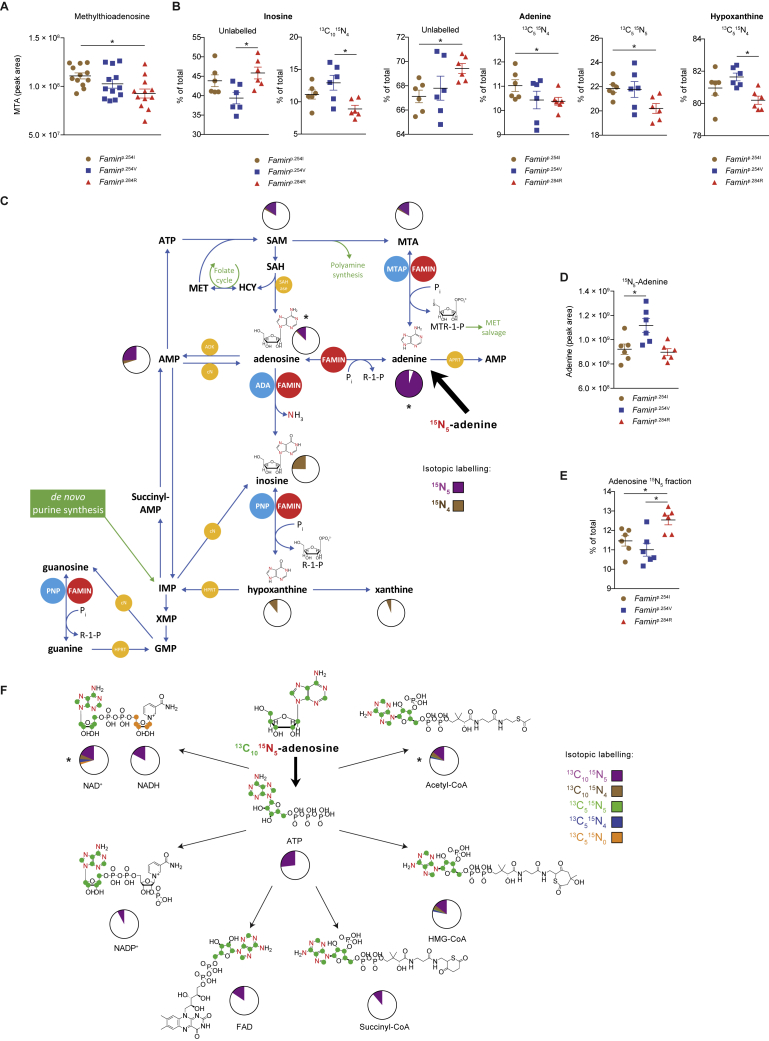
FAMIN Affects Routing through Central Purine Metabolism, Related to Figure 4 (A) Methylthioadenosine levels in *Famin*^p.254I^, *Famin*^p.254V^, *Famin*^p.284R^ M1 macrophages (n = 12). (B) Fraction of inosine, adenine and hypoxanthine labeled as the indicated isotopomer in M1 macrophages after a 3 h pulse with [^13^C_10_^15^N_5_] adenosine (n = 6). (C) Metabolic fate of stable isotope-labeled [^15^N_5_] adenine after a 3 h pulse of M1 macrophages (n = 6; mean). Schematic representation of central purine metabolism. AMP can be generated from adenine via sequential FAMIN and ADK activities or via APRT without loss of label (purple). Labeled [^15^N_5_] adenosine can be deaminated into inosine by FAMIN or ADA, with a loss of a single ^15^N as ammonia, generating a [^15^N_4_] isotopomer (brown). Routes of interconversion and relationship with other metabolic pathways are also illustrated. Fractions of different labeled states averaged across genotypes following the 3 h pulse with [^15^N_5_] adenine are depicted as pie charts; asterisks indicate metabolites with significantly altered isotopic labeling across genotypes as depicted in Figures S4D and S4E. Adenine phosphoribosyl transferase (APRT); adenosine kinase (ADK); adenosine deaminase (ADA); cytosolic nucleotidase (cN); hypoxanthine-guanine phosphoribosyl transferase (HPRT); purine nucleoside phosphorylase (PNP); *S*-methyl-5′-thioadenosine phosphorylase (MTAP); *S*-adenosylhomocysteine hydrolase (SAHase). (D) [^15^N_5_] adenine levels in M1 macrophages after a 3 h pulse with [^15^N_5_] adenine (n = 6). (E) Fraction of adenosine labeled as the [^15^N_5_] isotopomer in M1 macrophages after a 3 h pulse with [^15^N_5_] adenine (n = 6). (F) Fraction of ATP, NAD^+^, NADH, FAD, acetyl-CoA, HMG-CoA and succinyl-CoA labeled as the indicated isotopomer in M1 macrophages after a 3 h pulse with [^13^C_10_^15^N_5_] adenosine. Fractions of different labeled states (averaged across *Famin*^p.254I^, *Famin*^p.254V^ and *Famin*^p.284R^ genotypes) are depicted as pie charts. Asterisks indicate significantly altered isotopic labeling across *Famin* genotypes (n = 6 per genotype). Data are represented as mean ± SEM. ^∗^p < 0.05, ^∗∗^p < 0.01, ^∗∗∗^p < 0.001 (unpaired, two-tailed Student’s t test or one-way ANOVA).

**Figure S5:**
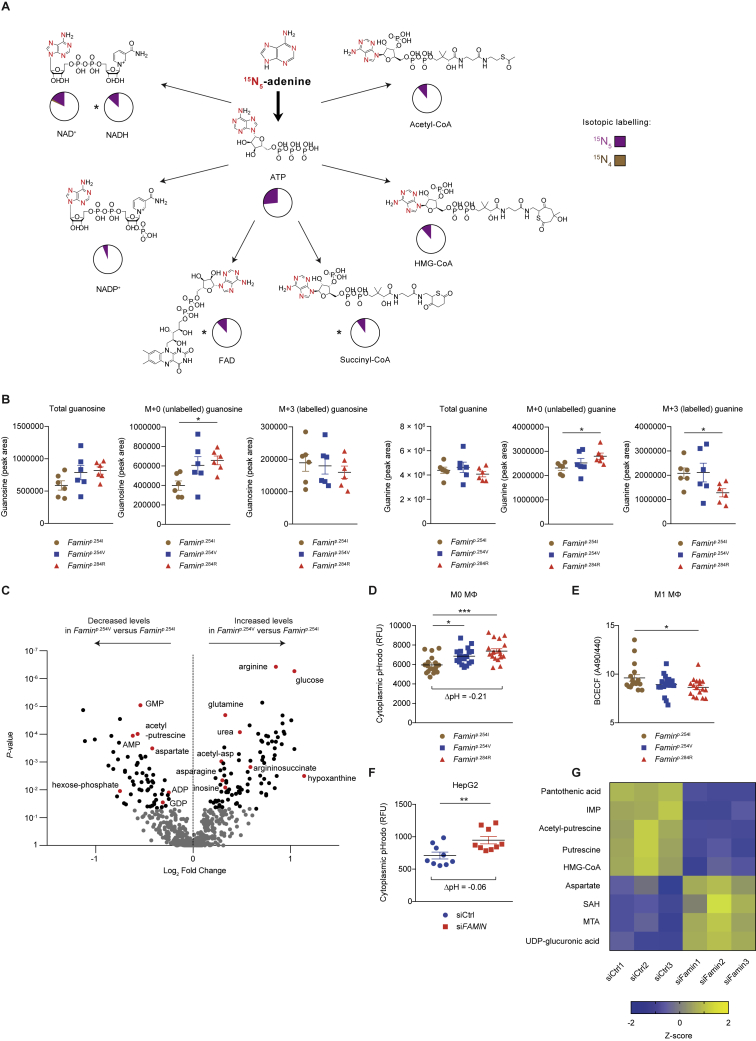
FAMIN Activity Affects Purine-Containing Cofactor Turnover, Related to Figure 4 (A) Fraction of ATP, NAD^+^, NADH, FAD, acetyl-CoA, HMG-CoA and succinyl-CoA labeled as the indicated isotopomer in M1 macrophages after a 3 h pulse with [^15^N_5_] adenine. Fractions of different labeled states (averaged across *Famin*^p.254I^, *Famin*^p.254V^ and *Famin*^p.284R^ genotypes) are depicted as pie charts. Asterisks indicate significantly altered isotopic labeling across *Famin* genotypes (n = 6 per genotype). (B) Total, unlabelled and [^13^C_1_^15^N_2_] guanine and guanosine levels in M1 macrophages after a 3 h pulse with [^13^C_1_^15^N_2_] guanosine (n = 6). (C) Differential metabolite levels in *Famin*^p.254I^ versus *Famin*^p.254V^ M1 macrophages. Data depicted as a volcano plot using p value and log_2_ fold change. Grey dots are non-significant, while black dots correspond to LCMS features with significantly altered abundance following Benjamini-Hochberg correction for multiple testing. Differential metabolites shown in red were confirmed and identified as indicated (n = 6). (D) Cytoplasmic pH measured using pHrodo in *Famin*^p.254I^, *Famin*^p.254V^, *Famin*^p.284R^ M0 macrophages (n = 18) (E) Cytoplasmic pH measured using BCECF with dual excitation at 440nm and 490nm in *Famin*^p.254I^, *Famin*^p.254V^, *Famin*^p.284R^ M1 macrophages (n = 18). Reduced 490:440 ratio corresponds to lower (more acidic) pH_c_. (F) Cytoplasmic pH measured using pHrodo in control and *FAMIN*-silenced HepG2 cells 48 h after transfection (n = 9). (G) Heatmap of metabolites in control and *FAMIN*-silenced HepG2 cells 24 h after transfection (n = 3). Data are represented as mean ± SEM. ^∗^p < 0.05, ^∗∗^p < 0.01, ^∗∗∗^p < 0.001 (unpaired, two-tailed Student’s t test or one-way ANOVA).

We next turned to murine bone marrow (BM)-derived M1 macrophages, in which disease-linked *Famin* variants cause immunometabolic compromise (Cader et al., 2016). In contrast to HepG2 cells, macrophages only use purine nucleotide salvage and hardly any *de novo* synthesis (Figures S3K–S3O). ADA, PNP, and MTAP expression is not affected by *Famin* genotype (Figure S3P). Cellular levels of adenine, adenosine, and ATP were lowest in *Famin*^p.284R^, intermediate in *Famin*^p.254V^, and highest in *Famin*^p.254I^ macrophages (Figures 4C and 4D). MTA followed a similar pattern (Figure S4A). Hence, FAMIN increased the availability of adenyl groups, from nucleobase to nucleotide triphosphate. After a 3-h pulse with [^13^C_10_^15^N_5_] adenosine, 14% of cellular adenosine was of the [^13^C_10_^15^N_5_] isotopomer, irrespective of *Famin* genotype (Figure 4E). An even larger fraction of adenine, AMP, SAM cycle, and purine salvage metabolites had label incorporated (Figure 4E). They featured adenyl groups that had undergone deamination (i.e., ^15^N_5_→^15^N_4_) and/or phosphorolysis (i.e., ^13^C_10_→^13^C_5_) (Figure 4E). Fractional incorporation of ^13^C and ^15^N into inosine, adenine, and hypoxanthine exhibited significant, although modest, differences across *Famin* genotypes (Figure S4B; Table S6). By tracing a 3-h [^15^N_5_] adenine pulse, we found that less than 6% of cellular adenine remained unlabeled (Figure S4C) and 12% of adenosine was of the [^15^N_5_] isotopomer, exhibiting genotype-specific differences (Figures S4C–S4E; and Table S6). After pulsing with either adenosine or adenine, one-fourth to one-third of AMP and ATP had label incorporated (Figures 4E and S4C). This high adenyl turnover extended to cofactors, i.e., coenzyme A (CoA), SAM, NAD^+^, NADP, and flavin adenine dinucleotide (FAD) (Figures S4F and S5A), again with *Famin* genotype-specific differences (Table S6). Adenyl metabolism is highly interconnected including fast substrate cycles (Boison, 2013). Exogenous adenosine or adenine would not directly contact FAMIN without prior metabolization, potentially obscuring the extent of FAMIN’s contribution. We, therefore, traced [^13^C_1_^15^N_2_] guanosine, which has fewer metabolic fates. After a 3-h pulse, fractional incorporation of [^13^C_1_^15^N_2_] increased from 19.5% ± 1.6% in *Famin*^p.284R^ to 31.9% ± 1.3% in *Famin*^p.254I^ macrophages (Figure 4F), driven by unlabeled guanosine accumulating in *Famin*^p.284R^ cells (Figure S5B; Table S6). Similarly, the fraction of [^13^C_1_^15^N_2_]-labeled guanine increased from 30.9% ± 3% in *Famin*^p.284R^ to 46.8% ± 2.6% in *Famin*^p.254I^ macrophages (Figure 4G), whereas total levels of guanine remained constant across genotypes (Figure S5B). Fractional labeling of guanosine triphosphate (GTP) was also higher in *Famin*^p.254I^ than in *Famin*^p.254V^ and *Famin*^p.284R^ macrophages (Figure 4H). Hence, FAMIN activity accounted for >50% higher fractional guanosine and guanine and >30% higher GTP labeling compared to cells without FAMIN activity. This demonstrated that FAMIN profoundly affected purine metabolism.

### FAMIN Prevents Cytoplasmic Acidification

**Figure 5:**
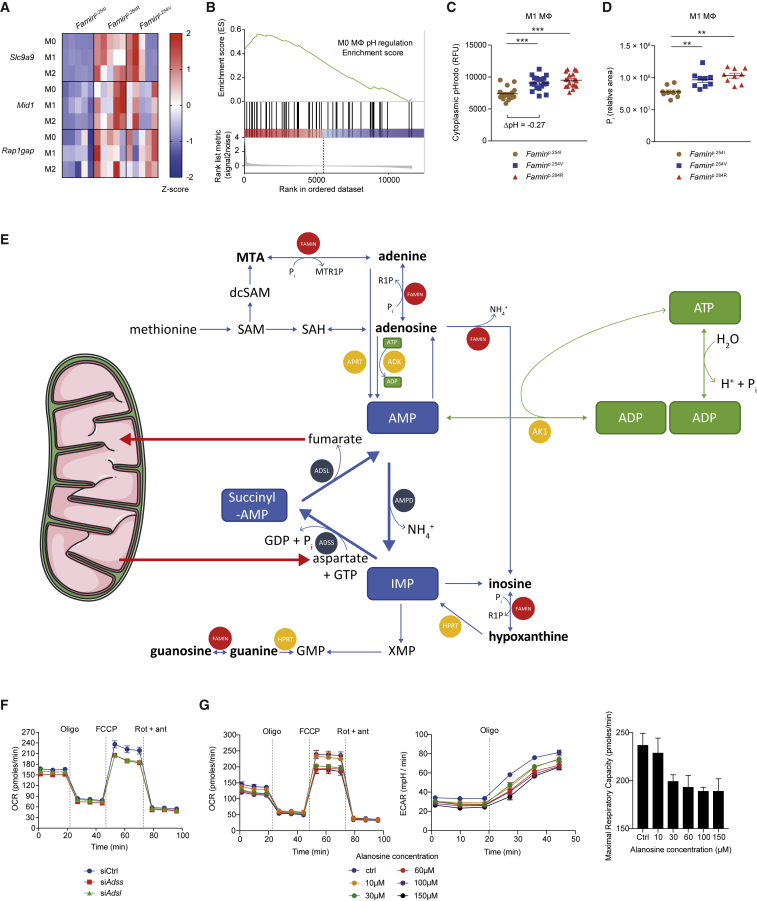
FAMIN Activity Controls Cellular pH and Enables a Purine Nucleotide Cycle (A) Heatmap of differentially expressed genes by *Famin* genotype in M0, M1, and M2 macrophages (n = 5). (B) Gene set enrichment analysis in *Famin*^p.254I^ compared to *Famin*^p.284R^ M0 macrophage transcriptomes; Gene Ontology pH regulation gene set (n = 5). (C) Cytoplasmic pH measured by pHrodo in M1 macrophages (n = 18). (D) Inorganic phosphate levels in M1 macrophages (n = 9). (E) Schematic of the purine nucleotide cycle (PNC; blue boxes and circles) with phosphorolysis, deamination, and salvage routes involving FAMIN. AMPD, AMP deaminase; ADSS, adenylosuccinate synthase; ADSL, adenylosuccinate lyase; AK1, adenylate kinase. (F) Oxygen consumption rate (OCR) of *Famin*^p.254I^ M0 macrophages silenced for *Adsl* or *Adss* or transfected with siRNA control. Basal OCR followed by (dotted vertical lines) oligomycin A (Oligo), FCCP, and rotenone plus antimycin A (Rot + ant) (n = 3). (G) OCR (left), extracellular acidification rate (ECAR) (middle), and maximal respiratory capacity (right) of *Famin*^p.254I^ M0 macrophages treated with *L*-alanosine. (n = 3). Data represented as mean ± SEM. ^∗^p < 0.05, ^∗∗^p < 0.01, and ^∗∗∗^p < 0.001 (one-way ANOVA).

How does purine metabolism by FAMIN control glycolysis and OXPHOS? Unbiased high-resolution LC-MS of M1 macrophage aqueous extracts revealed 67 LC-MS features of differential abundance between *Famin*^p.254I^ and *Famin*^p.284R^ and 157 between *Famin*^p.254I^ and *Famin*^p.254V^ cells after false discovery rate (FDR) correction (Figures 4I and S5C). Reassuringly, top dysregulated metabolites were adenine, inosine, hypoxanthine, ADP, AMP, and guanosine monophosphate (GMP) (Figures 4I and S5C). Key metabolites of glycolysis, FAO, amino acid and polyamine metabolism, and the urea cycle were among non-purines dysregulated by FAMIN variants (Figures 4I and S5C). The energetic compromise (Figure 4D; Cader et al., 2016) did not lead to increased phosphorylation of AMP-activated protein kinase (AMPK), a key energy sensor (Lin and Hardie, 2018), in *Famin*^p.284R^ compared to *Famin*^p.254I^ macrophages (Figure 4J), consistent with AMP being reduced in parallel with ATP (Figure 4I). Massively parallel RNA sequencing of M0, M1, and M2 macrophages revealed only *Slc9a9*, *Mid1*, and *Rap1gap* as differentially expressed in *Famin*^p.254V^ compared to *Famin*^p.254I^, and only a further 32 transcripts as different between *Famin*^p.284R^ and *Famin*^p.254I^ (Figure 5A; Table S7). The near-absence of a transcriptional response to the cells’ immunometabolic compromise pointed to a bona fide biochemical mechanism. *Slc9a9*, which encodes the Na^+^-H^+^ transporter 9 (NHE9) (Slepkov et al., 2007), provided an important clue. Na^+^-H^+^ transporters dynamically protect against cytoplasmic acidification stemming from the generation of protons (H^+^) by metabolic reactions, primarily the hydrolysis of ATP into ADP, P_i_, and H^+^ (Casey et al., 2010, Ipata and Pesi, 2018, Robergs et al., 2004). The Gene Ontology set “pH regulation” was duly enriched in *Famin*^p.284R^ compared to *Famin*^p.254I^ macrophages (Figure 5B). Measuring cytoplasmic pH (pH_c_) revealed a more acidic cytoplasm in *Famin*^p.254V^ and *Famin*^p.284R^ than in *Famin*^p.254I^ M0 and M1 macrophages (Figures 5C and S5D). Acidification was confirmed by a dual-excitation ratiometric pH_c_ indicator that internally controls for probe uptake (Figure S5E) and extended to *FAMIN-* compared to control-silenced HepG2 cells (Figure S5F).

### A Purine Nucleotide Cycle Is Active in Macrophages

**Figure S6:**
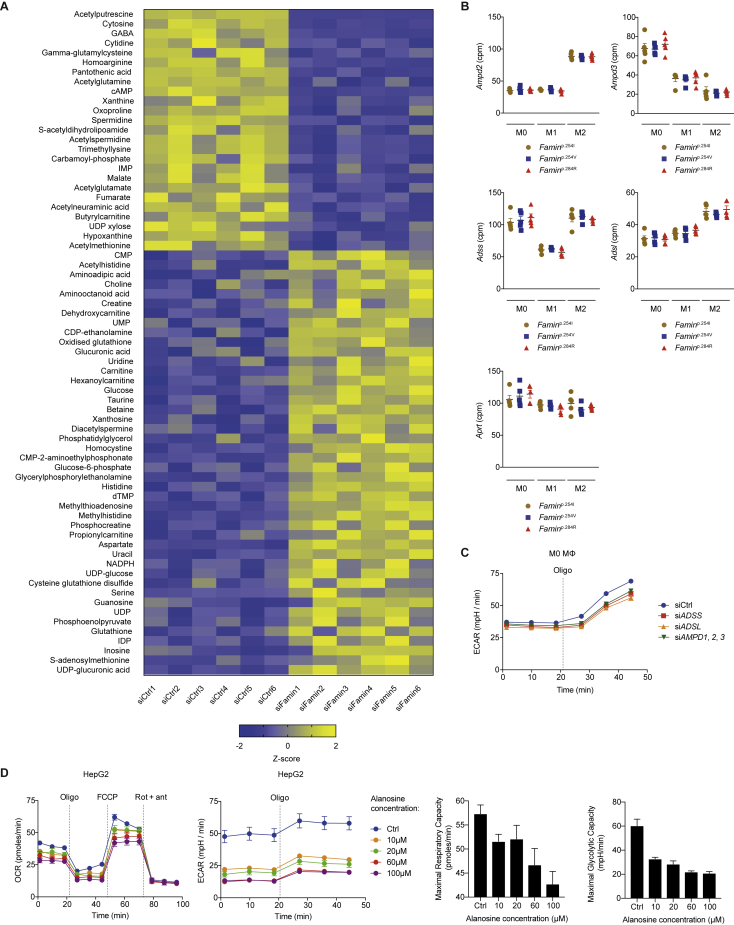
A Purine Nucleotide Cycle Operates in Macrophages and HepG2 Cells, Related to Figure 5 (A) Heatmap of metabolites in control and *FAMIN*-silenced HepG2 cells 48 h after transfection (n = 6). (B) *Ampd2*, *Ampd3*, *Adss*, *Adsl* and *Aprt* expression in *Famin*^p.254I^, *Famin*^p.254V^, *Famin*^p.284R^ M0, M1 and M2 macrophages (n = 5). *Ampd*: AMP deaminase; *Adss*: Adenylosuccinate synthase; *Adsl*: Adenylosuccinate lyase; *Aprt*: Adenine phosphoribosyltransferase. (C) Extracellular acidification rate (ECAR) of *Famin*^p.254I^ M0 macrophages silenced for *Adss, Adsl and Ampd*s *(Ampd1, 2 and 3)* or transfected with a non-targeting scrambled siRNA control. Basal ECAR measurement was followed by sequential treatment (dotted vertical lines) with oligomycin A (Oligo) (n = 3). *Ampd*: AMP deaminase; *Adss*: Adenylosuccinate synthase; *Adsl*: Adenylosuccinate lyase. (D) Oxygen consumption rate (OCR), and extracellular acidification rate (ECAR) with maximal respiratory and glycolytic capacities of control and *FAMIN*-silenced HepG2 cells treated with 10 μM, 20 μM, 60 μM, 100 μM of *L*-alanosine or vehicle control for 24 h. Basal OCR and ECAR measurements followed by sequential treatment (dotted vertical lines) with oligomycin A (Oligo), FCCP, and rotenone plus antimycin A (Rot + ant) (n = 3). Data are represented as mean ± SEM.

Cytoplasmic acidosis in *Famin*^p.254V^ and *Famin*^p.284R^ compared to *Famin*^p.254I^ M1 macrophages was accompanied by elevated levels of cellular P_i_ (Figure 5D). *Famin*^–/–^ macrophages are also depleted of phosphocreatine (Cader et al., 2016), a phosphoryl group carrier for near-immediate ADP rephosphorylation. Cytoplasmic acidosis, P_i_ accumulation, dwindling ATP levels, and depletion of phosphocreatine are hallmarks of exhaustive muscle exercise (Ipata and Pesi, 2018, Robergs et al., 2004). They occur when the rate of ATP hydrolysis is higher than fast-paced ADP rephosphorylation by glycolysis and phosphocreatine, hence, when the capacity to recycle H^+^ by creatine kinase, lactate dehydrogenase (LDH), and (slow) mitochondrial uptake is exceeded. When immediate rephosphorylation is impossible, the myokinase reaction (adenylate kinase, AK1) converts two molecules of ADP into one ATP and one AMP (Figure 5E; Ipata et al., 2011). In muscle cells, AMP enters the cytoplasmic purine nucleotide cycle (PNC) consisting of AMP deaminase (AMPD), adenylosuccinate synthase (ADSS), and adenylosuccinate lyase (ADSL) (Ipata and Pesi, 2018, Lowenstein and Tornheim, 1971, Lowenstein, 1990). AMPD releases NH_4_^+^ and generates IMP; ADSS synthesizes succinyl-AMP from IMP and aspartate at the expense of GTP; and ADSL regenerates AMP by releasing fumarate, which after conversion to malate enters mitochondria (Figure 5E). A reduction of IMP and an increase in aspartate were among few early (24 h; Figure S5G) changes and a decrease in fumarate and malate among later (48 h; Figure S6A) changes after *FAMIN* siRNA transfection into HepG2 cells. Murine primary macrophages express *Ampd2*, *Ampd3*, *Adss*, and *Adsl* (Figure S6B). Knockdown of *Adss* or *Adsl* in *Famin*^p.254I^ M0 macrophages reduced baseline oxygen consumption rate (OCR) compared to controls (Figure 5F). Uncoupling respiration from mitochondrial ATP synthesis by carbonyl cyanide-4-phenylhydrazone (FCCP; i.e., eliciting SRC) revealed an even larger difference in OCR between *Adss* and *Adsl* compared to controls (Figure 5F). *L*-Alanosine is a potent inhibitor of ADSS, the rate limiting step of the PNC (Tyagi and Cooney, 1980, Tyagi and Cooney, 1984). It dose-dependently reduced baseline OCR and SRC in *Famin*^p.254I^ M0 macrophages (Figure 5G), without affecting non-mitochondrial respiration after antimycin A and rotenone inhibition of the electron transport chain (ETC) (Figure 5G). *L*-Alanosine also reduced baseline and oligomycin-elicited extracellular acidification rate (ECAR) in M0 macrophages (Figure 5G). Such reduction was also observed upon silencing *Adss*, *Adsl*, or the *Ampd*s (Figure S6C). *L*-Alanosine dose-dependently reduced OCR, SRC, and ECAR in HepG2 cells as well (Figure S6D). This coordinated reduction in OCR and ECAR demonstrated that the PNC was active in macrophages and HepG2 cells and controlled OXPHOS and glycolytic activity.

### Flux through the Purine Nucleotide Cycle Requires FAMIN

**Figure 6:**
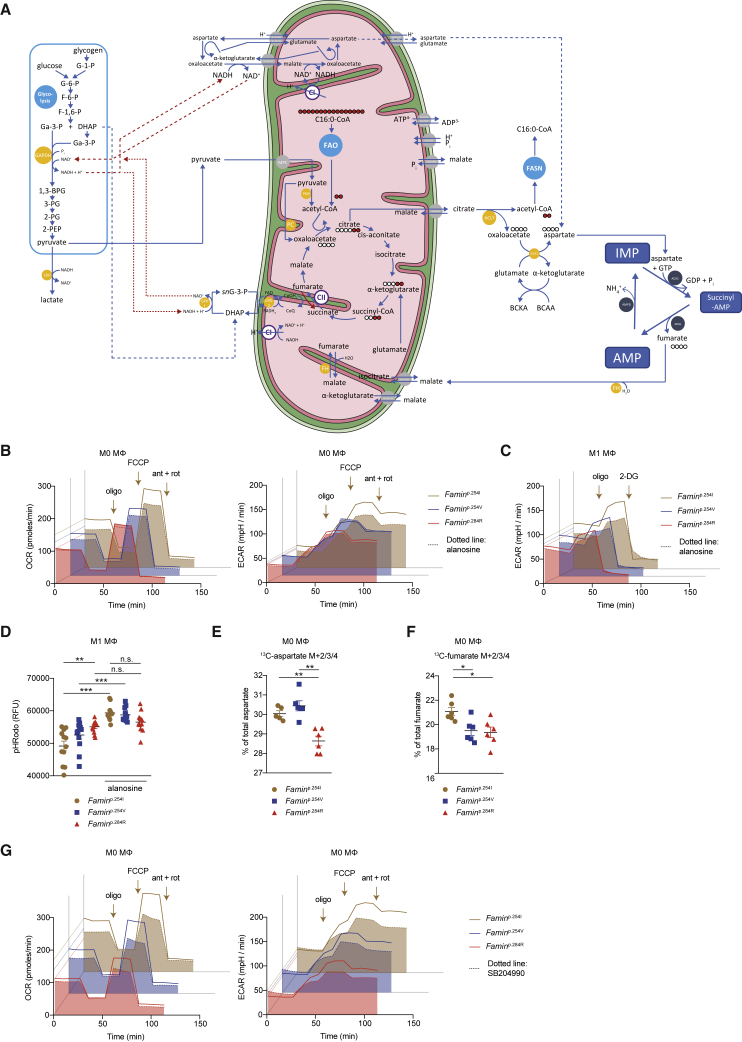
A FAMIN-Dependent PNC Controls Energy Metabolism (A) Schematic of cellular energy metabolism in context of the PNC. PNC enzymes (blue circles), other enzymes (yellow circles), and transporters (gray circles) with gene names. Electron transfer from glycolysis to mitochondria by the glycerol-3-phosphate (G3PS; connected with red dotted lines) and malate-aspartate shuttle (MAS; connected with red dashed lines). Filled red circles depict fate of [^13^C_16_] palmitic acid (C16:0)-derived carbons through fatty acid oxidation (FAO) into tricarboxylic acid (TCA) cycle citrate and by ATP citrate lyase (ACLY) into fatty acid synthesis (FASN); empty circles depict route of carbons from TCA oxaloacetate; labeling of α-ketoglutarate and succinyl-CoA depicted for M0 macrophages with intact TCA cycle. Complex II (CII) forward and reverse activity, blue and red arrowed arcs, respectively. CoQ, coenzyme Q; CI, complex I; branched chain amino and keto acids (BCAA, BCKA); DHAP, dihydroxyacetone phosphate; G-6-P, glucose-6-phosphate; F-6-P, fructose-6-phosphate; F-1,6-P, fructose-1,6-bisphosphate; Ga-3-P, glyceraldehyde-3-phosphate; 1,3-BPG, 1,3-bis-phosphoglycerate; 3-PG, 3-phosphoglycerate; 2-PG, 2-phosphoglycerate; 2-PEP, 2-phosphoenolpyruvate. (B) OCR (left) and ECAR (right) of M0 macrophages treated (dotted line + fill) with *L*-alanosine for 3 h or vehicle (solid line). Basal OCR and ECAR followed by (arrows) oligomycin A (Oligo), FCCP, and rotenone plus antimycin A (Rot + ant) (n = 3). (C) ECAR of M1 macrophages treated (dotted line + fill) with *L*-alanosine or vehicle (solid line). Basal ECAR followed by (arrows) Oligo and 2-deoxyglucose (2-DG) (n = 3). (D) Cytoplasmic pH (pH_c_) measured using pHrodo in M1 macrophages treated with *L*-alanosine or vehicle (n = 12 from 3 mice per genotype). (E and F) Fraction of aspartate (E) or fumarate (F) labeled as the indicated isotopomer in M0 macrophages after a [^13^C_16_] palmitate pulse (n = 6). (G) OCR (left) and ECAR (right) of M0 macrophages treated (dotted line + fill) with SB204990 (ACLY inhibitor) or vehicle (solid line). (n = 3). Data represented as mean ± SEM. ^∗^p < 0.05, ^∗∗^p < 0.01, and ^∗∗∗^p < 0.001 (one-way ANOVA).

During exhaustive muscle contraction, a fraction of IMP generated in the PNC may enter an “oxypurine” cycle through dephosphorylation to inosine (Figures 5E and 6A; Ipata and Pesi, 2018). By the oxypurine cycle, the purine ring may re-enter the PNC after salvage by PNP and HPRT, which is thought to retain the purine ring pool as nucleosides and nucleobases could efflux. FAMIN could support such a substrate cycle for inosine and additionally provide an entry point for AMP-derived adenosine with salvage routes by HPRT or directly by APRT (Figures 5E and 6A) and, thereby, promote flux through the PNC. As reported (Cader et al., 2016), baseline OCR, SRC, and ECAR were highest in *Famin*^p.254I^, intermediate in *Famin*^p.254V^, and lowest in *Famin*^p.284R^ M0 macrophages (Figure 6B). *L*-Alanosine reduced levels of OCR, SRC, and ECAR in *Famin*^p.254I^ and *Famin*^p.254V^ M0 macrophages to levels in *Famin*^p.284R^ cells, while not further reducing them in the latter (Figure 6B). *L*-Alanosine also curtailed ECAR in M1 macrophages, again only in those with active FAMIN (Figure 6C). Despite reducing glycolysis, *L*-alanosine acidified the cytoplasm in *Famin*^p.254I^ and *Famin*^p.254V^ M1 macrophages to the level of *Famin*^p.284R^ cells, where *L*-alanosine did not further lower the pH_c_ (Figure 6D). Hence, FAMIN activity was essential for a PNC to ensue, which, in turn, controlled rates of OXPHOS and glycolysis and set the pH_c_.

### FAO and ACLY Facilitate the FAMIN-Dependent PNC in Macrophages

**Figure S7:**
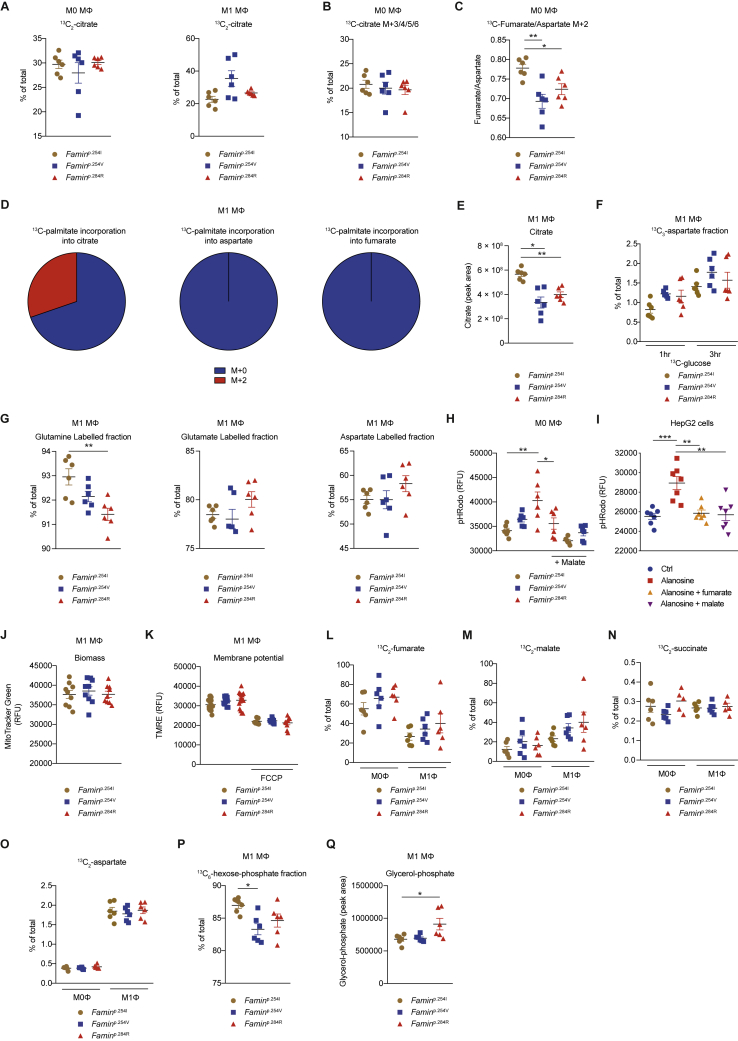
The Purine Nucleotide Cycle Is Linked to FAO, and FAMIN Deficiency Can Be Rescued by Exogenous Fumarate, Related to Figures 6 and 7 (A and B) Fraction of citrate labeled as the indicated isotopomer in *Famin*^p.254I^, *Famin*^p.254V^, *Famin*^p.284R^ M0 and M1 macrophages after a 3 h pulse with 100 μM [^13^C_16_] palmitate conjugated with BSA at 6:1 ratio (n = 6). (C) Ratio of ^13^C_2_-fumarate (M+2) to ^13^C_2_-aspartate (M+2) levels in M0 macrophages after a 3 h pulse with 100 μM [^13^C_16_] palmitate (n = 6). (D) Fractions of different labeled states of citrate, aspartate and fumarate (averaged across *Famin*^p.254I^, *Famin*^p.254V^ and *Famin*^p.284R^ genotypes) in M1 macrophages after a 3 h pulse with 100 μM [^13^C_16_] palmitate conjugated with BSA at 6:1 ratio (n = 6) depicted as pie chart. (E) Citrate levels in *Famin*^p.254I^, *Famin*^p.254V^, *Famin*^p.284R^ M1 macrophages (n = 6). (F) Fraction of aspartate labeled as the indicated isotopomer in *Famin*^p.254I^, *Famin*^p.254V^, *Famin*^p.284R^ M1 macrophages after a 1 or 3 h pulse with 2 g/L [^13^C_6_] glucose (n = 6). (G) Fraction of labeled glutamine, glutamate and aspartate in *Famin*^p.254I^, *Famin*^p.254V^, *Famin*^p.284R^ M1 macrophages after a 3 h pulse with 2 mM [^13^C_5_^15^N_2_] glutamine (n = 6). (H) Cytoplasmic pH (pH_c_) measured using pHrodo in *Famin*^p.254I^, *Famin*^p.254V^, *Famin*^p.284R^ M0 macrophages treated with 300 μM of malate or vehicle control for 24 h. Same control group as Figure 7D (n = 6). (I) Cytoplasmic pH (pH_c_) measured using pHrodo in HepG2 cells treated with vehicle control or 100 μM of *L*-alanosine for 24 h, supplemented as indicated with 300 μM fumarate or malate (n = 7). (J) Mitochondrial biomass measured using MitoTracker Green in *Famin*^p.254I^, *Famin*^p.254V^, *Famin*^p.284R^ M1 macrophages (n = 6). (K) Mitochondrial membrane potential measured using TMRE in *Famin*^p.254I^, *Famin*^p.254V^, *Famin*^p.284R^ M1 macrophages, and collapsed following FCCP treatment as indicated (n = 9/6). (L–O) Labeling of (L) fumarate, (M) malate, (N) succinate and (O) aspartate in M0 and M1 macrophages after a [^13^C_2_] fumarate pulse (n = 6). (P) Fraction of hexose-phosphate labeled as the indicated isotopomer in *Famin*^p.254I^, *Famin*^p.254V^, *Famin*^p.284R^ M1 macrophages after a 1 h pulse with 2 g/L [^13^C_6_] glucose (n = 6). (Q) Glycerol-3-phosphate in *Famin*^p.254I^, *Famin*^p.254V^, *Famin*^p.284R^ M1 macrophages (n = 6). Data are represented as mean ± SEM. ^∗^p < 0.05, ^∗∗^p < 0.01, ^∗∗∗^p < 0.001 (unpaired, two-tailed Student’s t test or one-way ANOVA).

We next studied the aspartate supplying the PNC (Figure 6A). Uptake of [^15^N_2_] aspartate into M1 macrophages was negligible (data not shown), consistent with most cultured cells’ reliance on *de novo* synthesis (Birsoy et al., 2015). Aspartate derives its carbons from oxaloacetate and its nitrogen from glutamate. Oxaloacetate can be generated from (1) malate by cytosolic or mitochondrial malate dehydrogenase, (2) pyruvate by mitochondrial pyruvate carboxylase, and (3) citrate by cytosolic ATP citrate lyase (ACLY) (Birsoy et al., 2015). A portion of cellular ACLY and FASN, with which FAMIN interacts (Cader et al., 2016), tethers to peroxisomes (Hillebrand et al., 2012). Higher OXPHOS and glycolytic activity in *Famin*^+/+^ than *Famin*^–/–^ macrophages required FAO and fatty acid synthesis (Cader et al., 2016). FAO-derived acetyl-CoA provides two carbons that combine with oxaloacetate to mitochondrial citrate, while ACLY cleaves citrate exported to the cytoplasm into oxaloacetate and acetyl-CoA that supplies fatty acid synthesis (Figure 6A). After a 3-h [^13^C_16_] palmitate pulse, 20%–30% of citrate was of the [^13^C_2_] isotopomer in M0 and M1 macrophages (Figure S7A), confirming both oxidise fatty acids. [^13^C_16_] Palmitate-pulsed M0 cells also exhibited isotopomers with >2 carbons labeled (Figure S7B), i.e., citrate that had undergone at least one full oxidation cycle. This allowed tracing the oxaloacetate carbons (Figure 6A). Fractional incorporation into aspartate after the [^13^C_16_] palmitate pulse was substantial (∼30%) and higher in *Famin*^p.254I^ and *Famin*^p.254V^ than in *Famin*^p.284R^ M0 macrophages (Figure 6E; Table S6). It was also higher into fumarate in *Famin*^p.254I^ than in *Famin*^p.254V^ and *Famin*^p.284R^ M0 macrophages (Figure 6F; Table S6), with the fumarate:aspartate fractional labeling ratio highest in *Famin*^p.254I^ cells (Figure S7C). SB-204990, a selective ACLY inhibitor (Granchi, 2018), indeed lowered OCR and ECAR most markedly in *Famin*^p.254I^ and *Famin*^p.254V^ M0 macrophages (Figure 6G). No citrate isotopomers beyond [^13^C_2_] were observed in M1 macrophages (Figure S7D), consistent with a tricarboxylic acid (TCA) cycle break after citrate (Jha et al., 2015), and accordingly, no [^13^C] incorporation into aspartate and fumarate was detected (Figure S7D). Total citrate levels, however, were ∼2-fold higher in *Famin*^p.254I^ than in *Famin*^p.254V^ and *Famin*^p.284R^ M1 macrophages (Figure S7E). Altogether, these findings suggested that a seemingly futile cycle of FAO and fatty acid synthesis, also observed in other contexts (Yao et al., 2016), was involved in pulling TCA cycle oxaloacetate via ACLY into aspartate and through the PNC into fumarate.

Glucose was not a major carbon source for aspartate in M1 macrophages, with 1-h and 3-h [^13^C_6_] glucose pulses resulting in ∼1.5% [^13^C_3_] aspartate (Figure S7F; Table S6). Glutamine, by glutamate (Birsoy et al., 2015, Sullivan et al., 2015), was an important source of *de novo* aspartate carbon, but with [^13^C] aspartate after a 3-h [^13^C_5_^15^N_2_] glutamine pulse trending lower in *Famin*^p.254I^ than *Famin*^p.284R^ cells (Figure S7G; Table S6), it was unlikely to supply the PNC.

### Fumarate Rescues an Impaired PNC

**Figure 7:**
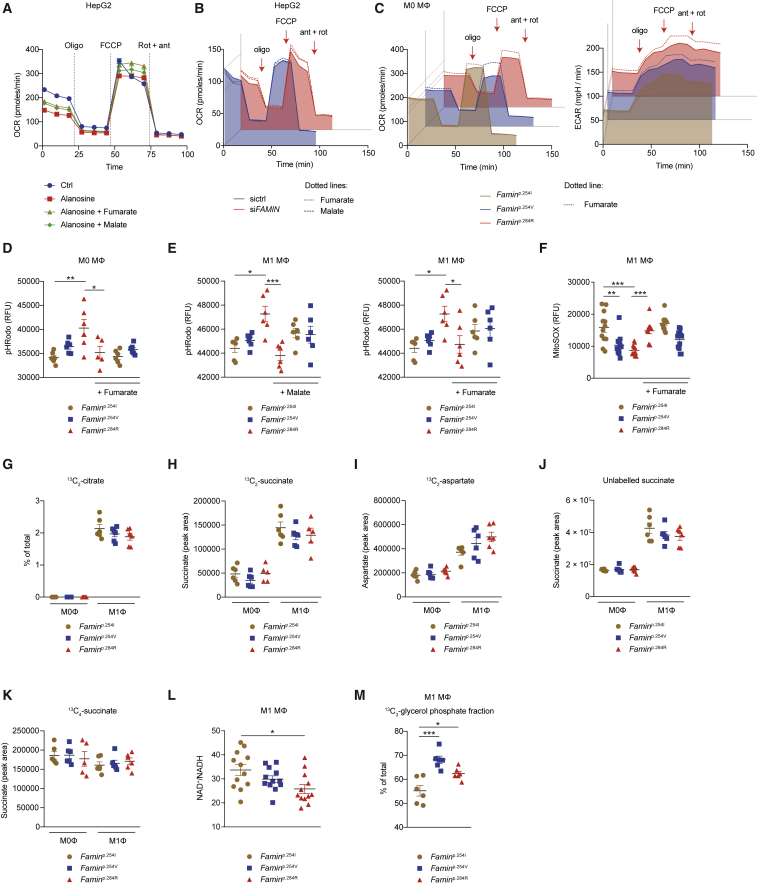
Fumarate Rescues the Impaired FAMIN-Dependent PNC (A) OCR of HepG2 cells treated with *L*-alanosine or vehicle and supplemented with fumarate or malate. Basal OCR followed by (dotted lines) Oligo, FCCP, and Rot + ant (n = 6). (B) OCR of control and *FAMIN*-silenced HepG2 cells treated with fumarate (dotted line), malate (dashed line), or vehicle control (solid line + fill). (n = 6). (C) OCR (left) and ECAR (right) of M0 macrophages treated with fumarate (dotted line) or vehicle (solid line + fill) for 8 h. (n = 3). (D and E) pH_c_ measured using pHrodo in M0 (D) or M1 macrophages (E) treated with malate, fumarate, or vehicle. Right and left panel of (E) share same control group (n = 6). (F) Mitochondrial superoxide measured using mitoSOX in M1 macrophages treated with fumarate or vehicle (n = 12). (G–J) Labeling of citrate (G), succinate (H, J, and K), and aspartate (I) in M0 and M1 macrophages after a [^13^C_2_] fumarate (G–I) or [^13^C_4_] fumarate pulse (J and K) (n = 6). (L) NAD^+^/NADH ratio in M1 macrophages (n = 12). (M) Fractional labeling of *sn*G-3-P in M1 macrophages after a [^13^C_6_] glucose pulse (n = 6). Data represented as mean ± SEM. ^∗^p < 0.05, ^∗∗^p < 0.01, and ^∗∗∗^p < 0.001 (one-way ANOVA).

We finally asked whether the metabolic compromise due to impaired FAMIN can be rescued. Fumarate and malate, which can be taken up by plasma membrane transporters (Pajor, 2014), increased baseline OCR and SRC in HepG2 cells with *L*-alanosine-blocked PNC (Figure 7A). Fumarate and malate partially rescued baseline OCR and SRC in *FAMIN*- but not control-silenced HepG2 cells (Figure 7B). Exogenous fumarate partially rescued OCR and SRC in *Famin*^p.254V^ and *Famin*^p.284R^ M0 macrophages but did not augment respiration in *Famin*^p.254I^ cells (Figure 7C). OXPHOS requires H^+^ and P_i_ import into the mitochondrial matrix. Fumarate and malate completely rescued cytoplasmic acidification in *Famin*^p.254V^ and *Famin*^p.284R^ M0 macrophages; they attained a pH_c_ equivalent to untreated *Famin*^p.254I^ cells, in which fumarate and malate did not affect pH_c_ (Figures 7D and S7H). Fumarate and malate similarly rescued the acidic pH_c_ in *L*-alanosine-treated HepG2 cells (Figure S7I) and, remarkably, also in *Famin*^p.284R^ M1 macrophages (Figure 7E), which are thought not to pursue OXPHOS (Mills et al., 2016). The mitochondrial ROS (mtROS) defect in *Famin*^p.254V^ and *Famin*^p.284R^ compared to *Famin*^p.254I^ M1 macrophages, present despite unaltered mitochondrial biomass and membrane potential (ΔΨ_m_) (Figures S7J and S7K), was also fully rescued by exogenous fumarate, whereas fumarate did not augment mtROS production in *Famin*^p.254I^ cells (Figure 7F). Importantly, fumarate also rescued the glycolysis defect of *Famin*^p.254V^ and *Famin*^p.284R^ M0 macrophages, as revealed by increased baseline and oligomycin-elicited ECAR but did not augment ECAR in *Famin*^p.254I^ cells (Figure 7C). Altogether, this was consistent with a FAMIN-dependent PNC sparking OXPHOS, increasing H^+^ uptake into mitochondria, and promoting glycolysis.

A 3-h [^13^C_2_] fumarate pulse, at the concentration that rescued the metabolic compromise, labeled ∼60% and ∼30% of cellular fumarate in M0 and M1 cells, respectively, resulting in ∼15% and ∼30% of cellular malate labeled (Figures S7L and S7M). Notably, [^13^C] did not incorporate into citrate in M0 and only labeled ∼2% of citrate in M1 macrophages (Figure 7G), excluding direct anaplerosis. We detected, however, [^13^C_2_] succinate in M1 and, surprisingly, also in M0 cells (Figures 7H and S7N) and [^13^C_2_] aspartate also in both macrophage types (Figures 7I and S7O). We confirmed the generation of [^13^C_4_] succinate from fully labeled [^13^C_4_] fumarate in both M1 and M0 macrophages after a 24-h pulse (Figures 7J and 7K). This implied fumarate accepted electrons from coenzyme Q (CoQH_2_) by a reverse succinate dehydrogenase (SDH; complex II) reaction, while conceivably increasing the capacity of the malate/aspartate shuttle (MAS). Reducing equivalents from glycolysis are imported into mitochondria by the MAS and the glycerol-3-phosphate shuttle (G3PS), thereby regenerating NAD^+^ required for the glyceraldehyde 3-phosphate dehydrogenase (GAPDH) reaction (Figure 6A; Robergs et al., 2004). The MAS transfers electrons to mitochondrial NAD^+^ before they enter the ETC, whereas the G3PS transfers them directly to CoQ (Mráček et al., 2013). The total cellular NAD^+^/NADH ratio was gradually diminished from *Famin*^p.254I^ by *Famin*^p.254V^ to *Famin*^p.284R^ M1 macrophages (Figure 7L). A 1-h [^13^C_6_] glucose pulse resulted in ∼90% of hexose-6-phosphate present as the [^13^C_6_] isotopomer in *Famin*^p.254I^ M1 macrophages, and this fraction was lower in *Famin*^p.254V^ and *Famin*^p.284R^ cells (Figure S7P), consistent with reduced glycolytic flux. At the same time, however, 55% of *sn*-glycerol-3-phosphate (*sn*G3P) exhibited [^13^C_3_] labeling in *Famin*^p.254I^ cells, and this was strikingly ∼10%–15% higher in *Famin*^p.254V^ and *Famin*^p.284R^ M1 macrophages (Figure 7M), which is indicative of a stalled GAPDH. *sn*G3P is the reduced form of the glycolysis intermediate dihydroxyacetone phosphate (DHAP) (Figure 6A), with which it forms the redox pair of the G3PS. *sn*G3P was among the top dysregulated metabolites in the unbiased survey (Figure 4I), with cellular levels higher in *Famin*^p.284R^ cells than the genotypes with active FAMIN (Figure S7Q). Altogether, this demonstrated that the FAMIN-enabled PNC balances the redox interface between glycolysis and the mitochondrial ETC by the fumarate it generates.

## Discussion

Here, we report FAMIN as a prototype of a new family of multifunctional purine enzymes conserved from bacteria to man. FAMIN combines adenosine phosphorylase activity, previously thought to be absent from eukaryotic metabolism, with activities analogous to ADA, PNP, and MTAP. The latter three enzymes had been considered the sole routes supplying purine nucleotide salvage, ADA and adenosine kinase (ADK) the only routes of adenosine conversion, and MTAP the sole route of adenine generation (Albers, 2009, Boison, 2013, Camici et al., 2018, Kamatani and Carson, 1981). FAMIN enables a PNC that controls pH_c_ and redox state and sets the pace of mitochondrial and glycolytic activity.

Adenine and ribose are considered primordial metabolites from which life emerged from prebiotic chemistry (Ralser, 2018). The combination of key activities of adenyl metabolism in one single enzyme is intriguing. FAMIN’s low catalytic rates appear disproportionate to its huge impact on cellular metabolism, possibly hinting to cofactors not present in our assays. Our YlmD structure identifies a single nucleoside-binding site, with the Cys^125^-His^80^-His^142^ triad, which is conserved across DUF152 proteins and FAMIN (residues Cys^284^, His^250^, and His^301^), coordinating the ribose moiety of inosine. The structures of DUF152 proteins suggest one single active site (Kim et al., 2006). The fold and active site architecture of YlmD and DUF152 are unrelated to those of purine and pyrimidine amidohydrolases, such as ADA, and of phosphorolytic enzymes (Appleby et al., 1999, Ealick et al., 1990, Kim et al., 2006, Wilson et al., 1991). A select few examples of enzymes have been described that catalyze at a single active site chemically distinct, physiologically utilized reactions. They were mostly discovered in bacteria with reduced genomes and may be reminiscent of long-extinct early cellular life that was likely much simpler, with few low activity enzymes performing multiple functions (Ferla et al., 2017, Say and Fuchs, 2010, Seelig, 2017). It is tempting to speculate that FAMIN and its antecedents may offer a glimpse into the ancient past, with its control over metabolic pace by redox state and pH. The pattern of FAMIN orthologs across species may suggest horizontal gene transfer. Orthologs are restricted to chordates within eukaryotes and to prokaryotic phyla typically present in the intestinal microbiota, an ecological niche of minuscule oxygen tension. The I254V variation is also an important difference between human and mouse, with C57BL/6 mice expressing *Famin*^p.254V^.

A PNC has not previously been reported in cells of the immune system. Lowenstein and colleagues first demonstrated a PNC, geared to stabilize an exercising muscle cell’s “energy charge” and releasing ammonia proportionate to work (Embden and Wassermeyer, 1928, Lowenstein and Tornheim, 1971, Lowenstein, 1972, Lowenstein, 1990, van Waarde, 1988). Inhibiting ADSS prevented the increase in TCA cycle intermediates during exercise, while not affecting levels at rest (Aragón and Lowenstein, 1980, Lowenstein, 1990, van Waarde, 1988). By funneling their amino nitrogen into the PNC, e.g., branched chain amino acids (BCAAs) become accessible to oxidation and carboxylation and, thereby, contribute to muscle anaplerosis (Arinze, 2005, Lowenstein, 1972, Lowenstein, 1990). The promotion of glycolysis has also been linked to the muscle PNC, albeit the mechanism remained unclear (Lowenstein, 1972, Sugden and Newsholme, 1975, van Waarde, 1988). A PNC in macrophages ensues only when FAMIN is active, as ADSS inhibition reduced OXPHOS, glycolysis, and pH_c_ to levels identical to those in FAMIN-deficient cells and no further in them. Interestingly, fumarate (and malate) rescued the acidic pH_c_ and the depressed mtROS production of FAMIN-deficient cells completely but the compromised OXPHOS and glycolysis only partially. No augmentation by exogenous fumarate occurred in cells with fully active FAMIN. Exogenous fumarate might not rescue processes feeding into aspartate entering the PNC, as exemplified by BCAA contributing to anaplerosis in muscle. The fumarate rescue itself entails substantial complexity, with mitochondrial import after hydration to malate by electroneutral transporters that exchange malate for α-ketoglutarate (part of the MAS), citrate (supplying ACLY), or phosphate (Palmieri, 2004, Palmieri, 2013) and electrogenic exchange of aspartate^–^ for glutamate^–^ + H^+^ (as part of the MAS). Mitochondrial H^+^ import occurs by the MAS and the G3PS, P_i_/H^+^ symport and cation exchange mechanisms (Robergs et al., 2004), hence, rectifying an imbalance in mitochondrial electron import by exogenous fumarate may directly rescue cytoplasmic acidosis.

Our data indeed suggest that PNC-supplied fumarate synchronizes mitochondrial activity with glycolysis by balancing electron transfer into mitochondria between the G3PS and MAS. Although both shuttles are likely affected, we favor a model where the primary redox perturbation in FAMIN-impaired macrophages arises from the G3PS. Specifically, glycolysis-derived electrons transferred by the G3PS to CoQ may be accepted by PNC-derived fumarate to form succinate (Figure 6A). In the absence of fumarate as (terminal) electron acceptor, mitochondrial superoxide production collapses, *sn*G3P accumulates, NADH is not recycled to NAD^+^, and GAPDH stalls. GPD2, the ETC *sn*G3P dehydrogenase, which is the rate-limiting enzyme of the G3PS, can generate high levels of mtROS (Mráček et al., 2013, Mráček et al., 2014), although mtROS could also arise from complex II or I (Chouchani et al., 2016, Langston et al., 2019, Mills et al., 2016). Such a model is particularly pertinent for M1 macrophages, which engage little OXPHOS with low (though not absent) oxygen consumption (Jha et al., 2015, Mills et al., 2016). Reverse SDH/complex II activity with succinate accumulation occurs in ischemic murine heart (Chouchani et al., 2014) and has been invoked for mtROS production in M1 macrophages (Mills et al., 2016), which is dependent on electron import by GPD2 (Langston et al., 2019). Succinate, complex II, and GPD2 are critical for interleukin-1β (IL-1β) secretion from M1 macrophages (Langston et al., 2019, Mills et al., 2016, Tannahill et al., 2013), a cytokine important in both CD and Still's disease (Ruperto et al., 2012, Uhlig and Powrie, 2018). Observing [^13^C_4_] succinate in [^13^C_4_] fumarate-pulsed M0 macrophages was surprising and controversially points toward a (small) fraction of SDH/complex II operating in reverse in parallel with its forward reaction in the TCA and ETC. Supporting fumarate respiration, present in many microaerophiles and anaerobes (Cook et al., 2014, Kröger et al., 1992), might be the evolutionary context of DUF152 proteins. It will be interesting to explore whether such parallel reverse SDH activity exists in mammalian cells and what the spatial basis is.

In summary, FAMIN and its orthologs rewrite core purine metabolism by revealing a surprising new layer of adenyl turnover. Even prior to this discovery, adenyl metabolism had been considered the most highly interconnected and exquisitely tuned metabolic circuitry in prokaryotes and eukaryotes.

## STAR★Methods

### Key Resources Table

**Table:** 

REAGENT or RESOURCE	SOURCE	IDENTIFIER
**Antibodies**		

PNP (H-7) (monoclonal)	Santa Cruz Biotechnology	Cat#sc-36508; RRID:AB_10845931
MTAP (42-T) (monoclonal)	Santa Cruz Biotechnology	Cat#sc-100782; RRID:AB_2147095
ADA (D-4) (monoclonal)	Santa Cruz Biotechnology	Cat#sc-28346; RRID:AB_626634
LACC1/FAMIN (E-12) (monoclonal)	Santa Cruz Biotechnology	Cat#sc-376231
ADA (polyclonal)	Novus Biologicals	Cat#NBP1-87404; RRID:AB_11025679
Beta-Actin, unconjugated, (13E5) (monoclonal)	Cell Signaling Technology	Cat#4970; RRID:AB_2223172
Beta-Actin, HRP Conjugated, (13E5) (monoclonal)	Cell Signaling Technology	Cat#5125; RRID:AB_1903890
Phospho AMPKα Thr172 (40H9) (monoclonal)	Cell Signaling Technology	Cat#2535; RRID:AB_331250
AMPKα (D5A2) (monoclonal)	Cell Signaling Technology	Cat#5831; RRID:AB_10622186

**Bacterial and Virus Strains**		

*E. coli:* BL21 (DE3) Competent Cells	Thermo Fisher Scientific	Cat#EC0114

**Biological Samples**		

N/A	N/A	N/A

**Chemicals, Peptides, and Recombinant Proteins**		

Recombinant human M-CSF	Peprotech	Cat#300-25
Ultrapure lipopolysaccharide (LPS) from *E. coli* K12	InvivoGen	Cat#tlrl-peklps
Recombinant murine IFN-γ	Peprotech	Cat#315-05
Oligomycin A	Sigma-Aldrich	Cat#75351; CAS:579-13-5
Carbonyl cyanide 4-(trifluoromethoxy)phenylhydrazone (FCCP)	Sigma-Aldrich	Cat#C2920; CAS:370-86-5
Rotenone	Sigma-Aldrich	Cat#R8875; CAS:83-79-4
Antimycin A from *Streptomyces* sp.	Sigma-Aldrich	Cat#A8674; CAS:1397-94-0
2-deoxy-*D*-glucose (2-DG)	Sigma-Aldrich	Cat#D8375; CAS:154-17-6
Adenosine	Sigma-Aldrich	Cat#A9251; CAS:58-61-7
Inosine	Sigma-Aldrich	Cat#I4625; CAS:58-63-9
Hypoxanthine	Sigma-Aldrich	Cat#H9377; CAS:68-94-0
5-Deoxy-5-(methylthio)adenosine (MTA)	Sigma-Aldrich	Cat#D5011; CAS:2457-80-9
*S*-(5-Adenosyl)-*L*-methionine	Sigma-Aldrich	Cat# A7007; CAS:86867-01-8
*S*-(5-Adenosyl)-*L*-homocysteine	Sigma-Aldrich	Cat#A9384; CAS:979-92-0
Cytidine	Sigma-Aldrich	Cat#C122106; CAS:65-46-3
Uridine	Sigma-Aldrich	Cat#U3750; CAS:58-96-8
2′-Deoxyadenosine	Sigma-Aldrich	Cat#D7400; CAS:16373-93-6
5′-Deoxyadenosine	Sigma-Aldrich	Cat#D1771; CAS:4754-39-6
Cholesterol Oxidase from *Streptomyces* sp.	Sigma-Aldrich	Cat#C8649; CAS:9028-76-6
*L*-Glutamine-^15^N_2_	Sigma-Aldrich	Cat#490032; CAS:204451-48-9
Palmitic acid-^13^C_16_	Sigma-Aldrich	Cat#605573; CAS:56599-85-0
Fumaric acid-^13^C_4_	Sigma-Aldrich	Cat#606014
Guanosine-^13^C_1_,^5^N_2_	Santa Cruz Biotechnology	Cat#sc-490348 CAS:197227-95-5
Sodium fumarate-2,3-^13^C_2_	Sigma-Aldrich	Cat#489468 CAS:287389-39-3
*L-*Glutamine-^13^C_5_,^15^N_2_	Sigma-Aldrich	Cat#607983
D-Glucose-^13^C	Sigma-Aldrich	Cat#389374; CAS:110187-42-3
Adenosine-^13^C_10_,^15^N_5_	Cambridge Isotope Laboratories	Cat#CNLM-3806 CA-PK
Adenine:HCL:1/2-H2O-^15^N_5_	Cambridge Isotope Laboratories	Cat#NLM-6924-PK
SB-204990 (ACLY inhibitor)	Cayman Chemical	Cat#15245;CAS: 154566-12-8
*L*-alanosine	Cayman Chemical	Cat#19545;CAS: 5854-93-3
Laccase from *Trametes versicolor*	Sigma-Aldrich	Cat#51639;CAS: 80498-15-3
ABTS	Sigma-Aldrich	Cat#10102946001; CAS:30931-67-0
Ferulic acid	Sigma-Aldrich	Cat#1270311;CAS:1135-24-6
Sinapic acid	Sigma-Aldrich	Cat#D7927; CAS:530-59-6
Fumaric acid	Sigma-Aldrich	Cat#F8509; CAS: 110-17-8
TRIzol Reagent	Thermo Fisher Scientific	Cat#15596026
Recombinant full-length human FAMIN (FAMIN^254I^ and FAMIN^254V^)	This paper	N/A
YfiH	This paper	N/A
YlmD	This paper	N/A
Recombinant truncated FAMIN (FAMIN^Δ176^)	This paper	N/A
TEV Protease	Sigma-Aldrich	Cat#T4455
*L*-Malic acid	Sigma-Aldrich	Cat#M7397; CAS: 97-67-6
Phenylmethylsulfonyl fluoride (PMSF)	Sigma-Aldrich	Cat#10837091001; CAS: 329-98-6
Hypoxanthine-^13^C_5_,^15^N_2_	Santa Cruz Biotechnology Ltd.	SC-353627
Adenosine monophosphate-^13^C_10_,^15^N_5_	Sigma Aldrich	Cat#650676
Adenosine triphosphate-^13^C_10_,^15^N_5_	Sigma Aldrich	Cat#710695
Succinic acid-^13^C_4_	Sigma Aldrich	Cat#491985
Cell Free Amino Acid Mixture- ^13^C,^15^N	Sigma Aldrich	Cat#767964-1EA

**Critical Commercial Assays**		

pHrodo Red AM Intracellular pH Indicator	Thermo Fisher Scientific	Cat#P35372
BCECF AM	Thermo Fisher Scientific	Cat#B1150
CyQUANT Cell Proliferation Assay Kit	Thermo Fisher Scientific	Cat#C35011
MitoSOX Red Mitochondrial Superoxide Indicator	Thermo Fisher Scientific	Cat#M36008
Mitochondrial Membrane Potential Assay Kit (TMRE)	Abcam	Cat#ab113852
MitoTracker Green FM	Thermo Fisher Scientific	Cat#M7514
Mouse Macrophage Nucleofector Kit	Lonza	Cat#VPA-1009
Lipofectamine RNAiMAX Transfection Reagent	Thermo Fisher Scientific	Cat#13778075
Pierce BCA Protein Assay Kit	Thermo Fisher Scientific	Cat#23225
Seahorse XFe96 FluxPak mini	Agilent	Cat#102601-100

**Deposited Data**		

RNA-Seq	This paper	GEO: GSE126641
Apo YlmD	This paper	PDB: 6T0Y
Inosine-bound YlmD	This paper	PDB: 6T1B

**Experimental Models: Cell Lines**		

HepG2	ATCC	Cat#HB-8065; RRID:CVCL_0027
Human Embryonic Kidney 293T (Hek293T)	ATCC	Cat#CRL-3216; RRID:CVCL_0063

**Experimental Models: Organisms/Strains**		

Mouse: *Famin*^p.284R^	(Cader et al., 2016)	N/A
Mouse: *Famin*^p.254I^	(Cader et al., 2016)	N/A
Mouse: *Famin*^p.254V^	(Cader et al., 2016)	N/A
Mouse: *Famin*^+/+^	(Cader et al., 2016)	N/A
Mouse: *Famin*^–/–^	(Cader et al., 2016)	N/A

**Oligonucleotides**		

siRNA targeting: human FAMIN (*LACC1*; *C13orf31*)	Horizon Discovery (Dharmacon)	Cat#M-015653-00
siRNA targeting: human purine nucleoside phosphorylase (*PNP*)	Horizon Discovery (Dharmacon)	Cat#M-009579-02
siRNA targeting: human methylthioadenosine phosphorylase (*MTAP*)	Horizon Discovery (Dharmacon)	Cat#M-009539-01
siRNA targeting: human adenosine deaminase (*ADA*)	Horizon Discovery (Dharmacon)	Cat#M-009588-01
siRNA targeting: mouse adenylosuccinate lyase (*ADSL*)	Horizon Discovery (Dharmacon)	Cat#L-064380-01
siRNA targeting: mouse adenylosuccinate synthase (*ADSS*)	Horizon Discovery (Dharmacon)	Cat#L-060265-01
siRNA targeting: mouse adenosine monophosphate deaminase 1 (*AMPD1*)	Horizon Discovery (Dharmacon)	Cat#L-048694-01
siRNA targeting: mouse adenosine monophosphate deaminase 2 (*AMPD2*)	Horizon Discovery (Dharmacon)	Cat#L-063716-01
siRNA targeting: mouse adenosine monophosphate deaminase 3 (*AMPD3*)	Horizon Discovery (Dharmacon)	Cat#L-010174-00

**Recombinant DNA**		

pESG-IBA105	IBA life sciences	Cat#5-4505-001
pPSG-IBA105	IBA life sciences	Cat#5-4305-001
pMAL-C5X	New England Biolabs	Cat# N8108S

**Software and Algorithms**		

Prism 8.0	GraphPad software	https://www.graphpad.com/; RRID:SCR_002798
Microsoft Excel	Microsoft	https://www.microsoft.com/en-gb/ RRID:SCR_016137
Thermo Xcalibur 4.1	Thermo Fisher Scientific	Cat#OPTON-30382; RRID:SCR_014593
Compound Discoverer 2.1	Thermo Fisher Scientific	Cat#OPTON-30834
Adobe Illustrator CC 2019 (23.0.3)	Adobe Inc.	https://www.adobe.com/products/illustrator.html; RRID:SCR_010279

**Other**		

Dextrin Sepharose MBPTrap High Performance column	GE Healthcare	Cat#28-9187-80
Q5 Site-Directed Mutagenesis Kit	New England BioLabs	Cat#E0554S
METLIN database	Scripps Research Institute	https://metlin.scripps.edu/landing_page.php?pgcontent=mainPage
The Universal Protein Resource (UniProt)	EMBL-EBI; Swiss Institute of Bioinformatics (SIB); Protein Information Resource (PIR) - Georgetown University	https://www.uniprot.org/help/about
Gravity flow Strep-Tactin XT Superflow	IBA GMBH	Cat#2-4012-001
PureYield Plasmid Maxiprep System	Promega	Cat#A2392
cOmplete Mini EDTA-free Protease Inhibitor Cocktail	Sigma-Aldrich	Cat#11836170001
Ni-NTA Superflow column	QIAGEN	Cat#30622
Superdex 200 Increase 10/300 GL	GE life sciences	Cat#28990944
Seahorse XF Base Medium	Agilent	Cat#102353-100
20 × LumiGLO Reagent	Cell Signaling Technology	Cat#7003
BEH amide (150 mm x 2.1 mm, 1.7 μm)	Waters Ltd.	Cat#186004802
BEH C8 (100 mm x 2.1 mm, 1.7 μm)	Waters Ltd.	Cat#186002878
ACE C18-PFP (150 mm x 2.1, 2 μm)	Hichrom	Cat#EXL-1010 1502U
Gemini NX-C18 (150 × 2 mm, 3 μm)	Phenomenex	Cat#00F-4453-B0

### Lead Contact and Materials Availability

Further information and requests for resources and reagents should be directed to and will be fulfilled by the Lead Contact, Arthur Kaser (ak729@cam.ac.uk). All unique/stable reagents generated in this study are available from the Lead Contact upon reasonable request but may require a completed Materials Transfer Agreement.

### Experimental Model and Subject Details

#### Mice

6- to 10-week-old mice were used for all experiments and were age- and gender-matched for individual experiments. C57BL/6N *Famin*^p.254I^, *Famin*^p.254V^, *Famin*^p.284R^, *Famin*^+/+^ and *Famin*^–/–^ mice have previously been described (Cader et al., 2016). Mice were heterozygously bred and for all experiments littermates were randomly assigned to experimental groups. Mice were maintained under specific pathogen-free conditions at the Central Biomedical Services facility, University of Cambridge. All procedures performed had local ethics and UK Home Office approval.

#### Cell lines

HepG2 (RRID: CVCL_0027), an adherent cell line isolated from liver lesions of a male Caucasian patient (ATCC HB-8065), and HEK293T (RRID: CVCL_0063) a cell line derived from human fetal kidney (ATCC CRL-3216) were maintained in DMEM medium and kept in a 5% carbon dioxide (CO_2_) incubator at 37°C. Unless otherwise stated, all culture media were supplemented with penicillin/streptomycin (1%) and heat-inactivated fetal bovine serum (10%). All cell lines used in this study were authenticated and purchased from ATCC and are not included within the register of misidentified cell lines curated by the International Cell Line Authentication Committee registry (ICLAC).

#### Murine bone marrow-derived macrophage

Bone marrow-derived macrophages (BMDMs) were prepared by flushing mouse femurs and tibias with PBS. Cells were filtered through a 70 μm cell strainer and re-suspended in complete RPMI-1640 medium (containing 100 U/mL of penicillin-streptomycin, 1 mM HEPES buffer and 10% FBS). To generate BMDMs, cells were cultured for 6 days in complete medium containing 100 ng/mL of M-CSF with media exchanged after 3 days. Macrophages were harvested, seeded and polarized for 24 h toward M1 macrophages with IFN-γ (50 ng/mL) plus LPS (20 ng/mL), or toward M2 macrophages with IL-4 (20 ng/mL), or left unstimulated and used as M0 macrophages. For adenosine, guanosine, adenine, fumarate and palmitate tracing experiments, M0 macrophages and/or M1 macrophages were pulsed for 3 h or as indicated with 50 μM [^13^C_10_^15^N_5_] adenosine, [^15^N_5_] adenine, [^13^C_1_^15^N_2_] guanosine or 100 μM [^13^C_16_] palmitate (6:1 conjugation with BSA) or 300 μM [^13^C_2_] fumarate, [^13^C_4_] fumarate prior to direct extraction with 4:1 methanol/H_2_O. For glucose tracing experiments, M1 macrophages were cultured for 1 h or as indicated in RPMI glucose-free medium supplemented with 2 g/L of [^13^C_6_] glucose prior to direct methanol extraction. For glutamine tracing experiments, M1 macrophages were cultured with RPMI-glutamine free medium supplemented with 2 mM of [^13^C_5_^15^N_2_] glutamine or [^15^N_2_] glutamine for 24 h prior to direct methanol extraction.

### Method Details

#### Plasmids

Complementary DNA encoding human FAMIN^254I^ (NP_001121775, NM_001128303) was cloned into a pESG-IBA105 vector (IBA Life Sciences) that contains an N-terminal Twin-Strep-tag and TEV cleavage motif. The FAMIN^254V^ construct was generated via site-directed mutagenesis using a Q5 Site-Directed Mutagenesis Kit (New England BioLabs), following manufacturer’s instructions. Sequences for YfiH (Uniprot P33644, PDB 1Z9T; from *Escherichia coli* strain K12, phylum *Proteobacteria*) and YlmD (Uniprot P84138, PDB 1T8H; from *Geobacillus stearothermophilus*, phylum *Firmicutes*) were synthesized by Origene and cloned into a pPSG-IBA105 vector (IBA Life Sciences). For FAMIN and FAMIN^Δ176^ (amino acids 176-430 of FAMIN) MBP fusion proteins, *FAMIN* cDNA sequences were synthesized by GenScript and codon-optimized for prokaryotic expression, and cloned into a pMAL-C5x (New England BioLabs) vector downstream of a MBP tag that is linked to Factor Xa and TEV protease cleavage motifs.

#### Mammalian expression and purification of FAMIN

HEK293T cells were transfected with PEI-DNA complex containing the Strep-tagged FAMIN expression plasmids. After 48-72 h, transfected cells were harvested in PBS and centrifuged twice at 800 × g for 3 min and the pellet resuspended in hypotonic lysis buffer (10 mM NaCl, 10 mM HEPES, 1 mM TCEP and 10% glycerol pH 7.6) supplemented with Complete Mini EDTA-free protease inhibitor cocktail (Sigma) plus PMSF at 1 mM. Following sonication and clearance by centrifugation at 50,000 × g, the lysates were then loaded onto a Streptactin XT Superflow column (IBA Life Sciences) pre-equilibrated with at least 4 column volumes of lysis buffer. The column was then washed with 10 column volumes of wash buffer (100 mM NaCl, 100 mM HEPES, 1 mM TCEP and 10% glycerol pH 7.6) and the Strep-tagged protein was eluted from the column with 6 column volumes of 50 mM biotin. The purified recombinant protein was then incubated with His-tagged TEV protease (Sigma) at a ratio of 10:1 as determined by A280 quantification using a NanoDrop spectrophotometer. The mixture was incubated overnight at 4°C. TEV protease was then removed by passing the mixture through a pre-equilibrated 1 mL Ni-NTA Superflow column (IBA Life Sciences). The flow-through constituting tag-free recombinant protein was collected. This was then concentrated with a 10 kDa column filter (Amicon) and further purified by size exclusion using an AKTA Superdex 200 increase (10/300) column (GE Life Sciences) column, followed by copious washing. Eluted fractions corresponding to positive peaks on the chromatogram were confirmed on Coomassie SDS-PAGE. Streptactin-purified FAMIN with retained tag exhibited activity indistinguishable from protein prepared, as described above, and was used in select experiments as indicated in the legend.

#### Prokaryotic expression and purification of MBP-FAMIN fusion proteins

*E. coli* BL21(DE3) cells were transformed with MBP-full length FAMIN or MBP-FAMIN^Δ176^ expression plasmids and grown at 30°C in ampicillin supplemented LB media. Expression was induced at an OD600 of 0.5 – 0.7 with 0.3 mM IPTG for 4 h. Cultures were harvested by centrifugation and cell pellets washed twice with PBS. Cells were then resuspended in 5 mL of lysis buffer containing 20mM NaCl, 20 mM HEPES, 10% glycerol, pH 8.0, 1× cOmplete Mini EDTA-free protease inhibitor cocktail (Roche). The cells were lysed by sonication on ice and cleared by centrifugation at 35,000× *g* for 30 minutes. The soluble fraction was treated with 5 μg / mL of DNase I (Sigma) and 10 μg / mL of RNase A (Sigma) for 15 minutes on ice. The protein-containing supernatant was filtered through a 0.2 µm filter, diluted in 10× volume of lysis buffer, and then loaded onto a pre-equilibrated dextrin sepharose MBPTrap High Performance column (GE Healthcare). The column was washed with 20 column volumes of modified lysis buffer containing 750 mM NaCl. The protein was eluted with 10 column volumes of a buffer containing 100 mM NaCl, 20 mM HEPES, 10 mM maltose, 10% glycerol, pH 8.0. The eluted fractions were pooled and concentrated for use in further assays.

#### Expression and Purification of YlmD and YfiH

*E. coli* BL21(DE3) cells transformed with the YlmD or YfiH expression plasmid were grown at 37°C in LB media containing 100 mg l^−1^ ampicillin. Expression was induced at an OD600 of 0.6 with 0.1 mM IPTG for 18 h at 18°C. Cultures were harvested by centrifugation at 7,000 × g then resuspended in lysis buffer containing 10 mM Tris, pH 8.0, 200 mM NaCl, 10% glycerol, 2 mM DTT, 1:10,000 (v/v) benzonase solution (Sigma), 1 × cOmplete Mini EDTA-free protease inhibitor cocktail (Roche). The cells were lysed by sonication on ice. The lysate was clarified by centrifugation (30 min, 35,000 × *g*). The protein-containing supernatant was filtered through a 0.45 μm filter and loaded onto a 5 mL StrepTrap column (GE Healthcare). The column was washed with 10 column volumes of 10 mM Tris, pH 8.0, 200 mM NaCl, 10% glycerol, 2 mM DTT. The protein was eluted using 4 column volumes of 10 mM Tris, pH 8.0, 200 mM NaCl, 10% glycerol, 2 mM DTT, 2.5 mM D-desthiobiotin. The elution fractions were pooled and further purified using on a Superdex 200 increase (10/300) size-exclusion column (GE Healthcare) in 10 mM HEPES pH 7.4, 200 mM NaCl, 0.5 mM TCEP.

#### Crystallization and crystallographic structure determination of YlmD

YlmD was concentrated to 5.6 mg ml^-1^ and crystal hanging drop vapor diffusion experiments were set up at 22°C in EasyXtal 15-well DG-tool plates (QIAGEN). Hanging drops were set up by mixing 1.5 μl of protein and 1.5 μl of reservoir solution, 0.1 M HEPES pH 7.5, 0.5 M NaCl, 25% PEG 6000. For data collection without inosine, crystals were harvested and soaked for 5-10 min in cryoprotectant containing 0.1 M HEPES pH 7.5, 0.5 M NaCl, 25% PEG 6000 and 20% glycerol. For data collection in the presence of inosine, crystals were soaked for 45-60 min in cryoprotectant containing 0.1 M HEPES pH 7.5, 0.5 M NaCl, 25% PEG 6000, 20% glycerol, 0.5 mM sodium phosphate, 0.5 mM zinc sulfate and 18.7 mM inosine. Overnight soaks with inosine and co-crystallization with inosine resulted in lower-resolution diffraction without improving electron density for bound inosine. The crystals were then flash frozen in liquid nitrogen. X-ray diffraction data were collected at 100 K at Diamond Light Source beamlines I03 and I04 and processed with autoPROC (Vonrhein et al., 2011) and STARANISO (Tickle et al., 2018).

The structure was determined by molecular replacement with Phaser using the available structure of YlmD (PDB 1T8H) as the search model (McCoy et al., 2007). Models were iteratively refined in Fourier space using data up to 1.2 Å resolution with REFMAC (Chen et al., 2010, Otwinowski and Minor, 1997) and PHENIX (Adams et al., 2010). Model building and real space refinement were performed in COOT version 0.8.9.2 (Emsley and Cowtan, 2004).

#### Extraction of aqueous metabolites

Cells were harvested as indicated using either direct extraction with 4:1 methanol:water or trypsin-EDTA (0.25%). Unless otherwise indicated, for direct methanol extraction, cells were washed twice with PBS followed by addition of pre-chilled 4:1 methanol:water. Cells were scraped and the resulting methanol mixture transferred to 2 mL-flat-bottomed screw cap tubes. Samples were then vortexed, sonicated and lastly centrifuged at 21,000 *g* for 10 minutes to pellet any debris. The supernatant was transferred to new 2 mL tubes for drying as described below. Measurement of cellular P_i_ levels necessitated phosphate-free buffer wash steps, with PBS replaced by a buffer consisting of 162mM ammonium acetate (pH 7.4). All solvents used were HPLC grade or higher and obtained from Honeywell (Fisher Scientific).

Cell pellets harvested using trypsinization were washed with PBS and then subjected to extraction using the methanol:chloroform method described by Folch et al. (1957). Briefly, a stainless steel ball (QIAGEN) was added to each washed cell pellet on dry ice along with 1 mL of ice cold 2:1 chloroform:methanol inside a 2 mL-flat-bottomed screw cap tube (Starlab). The samples were homogenized using a Tissue Lyser (QIAGEN) for 10 min at 25 Hz to ensure optimum extraction and ascertaining to freeze the tissue lyser plates prior to homogenization in order to keep samples cold during extraction. 400 μL of ice cold water was added and the samples thoroughly vortexed and sonicated for 5 minutes before centrifugation at 21,000 *g* for 5 min. After centrifugation the aqueous (top layer) and organic (bottom layer) fractions were separated and aliquoted into separate screw-cap tubes both kept on dry ice. A further 1 mL of 2:1 chloroform:methanol was added to the original tube containing the protein pellet and the extraction repeated as described above. The resulting layers were combined, dried (as described below) and stored at −20°C prior to further preparation and analysis.

#### LC-MS sample preparation

Aqueous extracts of cells or protein reaction mixtures were lyophilised using a centrifugal evaporator (Savant, Thermo Scientific) and reconstituted in 100 μL (for cell extracts) and 200 μL (for protein reaction mixtures) of 7:3 acetonitrile: 0.1 M aqueous ammonium carbonate containing 2 μM [^13^C_10_^15^N_5_] adenosine monophosphate, [^13^C_10_^15^N_5_] adenosine triphosphate, 10 μM [^13^C_4_] succinic acid and 10 μM [^13^C_5_^15^N_5_] glutamic acid (all from Sigma Aldrich except the glutamic acid from Cambridge Isotope Laboratories) as internal standards, although it should be noted that for experiments using labeled substrates, internal standards were omitted to avoid contamination of metabolites. The resulting solution was vortexed then sonicated for 15 min followed by centrifugation at 21,000 *g* to pellet any remaining undissolved material. Samples not run in HILIC mode were reconstituted directly with 50 μL of aqueous 10 mM ammonium acetate containing 2 μM [^13^C_10_^15^N_5_] adenosine monophosphate, adenosine triphosphate [^13^C_10_^15^N_5_], 10 μM [^13^C_4_] succinic acid and a 2 in 100000 dilution of a cell free amino acid mixture containing all the proteinogenic amino acids U[^13^C^15^N] labeled. Samples were thoroughly vortexed and sonicated as above. After centrifugation the supernatants were transferred with an automatic pipette into a 300 μL vial (Fisher Scientific) and capped ready for analysis. Ammonium carbonate and ammonium acetate were Optima grade obtained from Fisher Scientific.

#### LC-MS analysis of aqueous metabolites

For untargeted analysis, a Velos Pro Elite orbitrap mass spectrometer coupled to a U3000 chromatography system or a Q Exactive Plus orbitrap coupled to a Vanquish Horizon ultra high performance liquid chromatography system was used. For targeted analysis, samples were analyzed using a Quantiva triple stage quadrupole mass spectrometer coupled to a Vanquish Horizon (all analytical instrument combinations supplied by Thermo Fisher Scientific).

Samples were then analyzed using a bridged ethylene hybrid (BEH) amide hydrophilic interaction liquid chromatography (HILIC) approach for the highly polar aqueous metabolites. For this analysis the strong mobile phase (A) was 100 mM ammonium carbonate, the weak mobile phase was acetonitrile (B) with 1:1 water:acetonitrile being used for the needle wash. The LC column used was the BEH amide column (150 × 2.1 mm, 1.7 μm, Waters). The following linear gradient was used: 20% A in acetonitrile for 1.5 min followed by an increase to 60% A over 2.5 min with a further 1 min at 60% A after which the column was re-equilibrated for 1.9 min. After each chromatographic run the column was washed with 30 column volumes of 6:4 water:acetonitrile followed by a further 10 column volumes of 95:5 acetonitrile:water for storage. The total run time was 7 min, the flow rate was 0.6 mL/min and the injection volume was 5 μL. In order to resolve pentose phosphates for identification of ribose-1-phosphate a shallower gradient was employed: 30% A in acetonitrile for 2.0 minutes followed by an increase to 50% A over 3.0 minutes with re-equilibration for 1.9 minutes. After HILIC analysis samples were dried and reconstituted in the same volume of 10 mM ammonium acetate (for samples not run in HILIC mode this drying step was omitted) prior to orthogonal mixed mode analysis using an ACE Excel C18-PFP column (150 × 2.1 mm, 2.0 μm, Hichrom). Mobile phase A consisted of water with 10 mM ammonium formate and 0.1% formic acid and mobile phase B was acetonitrile with 0.1% formic acid. For gradient elution mobile phase B was held at 0% for 1.6 min followed by a linear gradient to 30% B over 4.0 minutes, a further increase to 90% over 1 min and a hold at 90% B for 1 min with re-equilibration for 1.5 minutes giving a total run time of 6.5 minutes. The flow rate was 0.5 mL/min and the injection volume was 2 μL. The needle wash used was 1:1 water:acetonitrile. Both chromatography modes were used for both targeted and untargeted analysis.

For basic pH reversed phase LC-MS analysis of analytes such as nucleoside phosphates and inorganic phosphate (P_i_; 96.9696 in negative ESI) in samples reconstituted in aqueous ammonium acetate, a base stable Phenomenex Gemini NX-C18 (150 × 2 mm, 3 μm) (Phenomenex Ltd., Macclesfield, UK) column was employed using the following chromatography method with a weak mobile phase (A) of 10 mM ammonium acetate with 0.1% ammonia and a strong mobile phase (B) of acetonitrile. Mobile phase B was held at 0% for 1.6 min, followed by a linear gradient to 30% B over 4.0 minutes, a further increase to 90% over 1 min, and a hold at 90% B for 1 min, with re-equilibration for 1.5 minutes giving a total run time of 6.5 minutes. The flow rate was 0.5 mL/min and the injection volume was 2 μL. The needle wash used was 1:1 water:acetonitrile.

For enzyme kinetic assays requiring quantitation of ribose-1-phosphate a further chromatographic approach was used to allow for analysis of all substrates and products using a single assay. A Waters BEH C8 column was used (100 × 2.1 mm, 1.7 μm) with a weak mobile phase (A) of aqueous 10 mM ammonium acetate with 0.1% ammonia and a strong mobile phase (B) of acetonitrile. For gradient elution mobile phase B was held at 0% for 1.6 min, followed by a linear gradient to 30% B over 4.0 minutes, a further increase to 90% over 1 min, and a hold at 90% B for 1 min, with re-equilibration for 1.5 minutes giving a total run time of 6.5 minutes. The flow rate was 0.5 mL/min and the injection volume was 2 μL. The needle wash used was 1:1 water:acetonitrile. For those assays requiring only nucleotide and nucleoside quantitation the acidic C18-PFP approach described above was used. The ammonium formate was Optima grade supplied by Fisher Scientific.

Untargeted analysis on the Elite used a high resolution FTMS full scan of 60-1500 *m/z* with a resolution of 60,000 ppm, where due to positive mode negative mode equilibration times each mode was run independently. Source parameters used for the orbitrap were a vaporizer temperature of 400°C, an ion transfer tube temperature of 300°C, an ion spray voltage of 3.5 kV (2.5 kV for negative ion mode) and a sheath gas, auxiliary gas and sweep gas of 55, 15 and 3 arbitrary units respectively with an S-lens RF (radio frequency) of 60%. For untargeted analysis using the Q Exactive Plus a full scan of 60-900 *m/z* was used at a resolution of 70,000 ppm where positive and negative ion mode assays were run separately in order to maximize data points across a peak at the chosen resolution. The source parameters were the same as those used for the Elite. For analysis of CoA species and reducing equivalents using the Q Exactive Plus orbitrap unique mass spectrometry methodology was employed where the full scan mass range was reduced to 500-1000 *m/z*, the capillary temperature was increased to 350°C and the S-lens RF to 100%.

Targeted analysis on the Quantiva utilized selected reaction monitoring (SRM) employing fast polarity switching with mass transitions and compound dependent parameters (collision energy voltage and RF lens voltage) determined on infusion of 1 μM standards at a flow rate of 10 μl/min in 4:1 acetonitrile:water with 0.1% acetic acid. Source parameters used were a vaporizer temperature of 440°C and ion transfer tube temperature of 362°C, an ion spray voltage of 3.5 kV (2.5 kV for negative ion mode) and a sheath gas, auxiliary gas and sweep gas of 54, 17 and 2 arbitrary units respectively. All fragmentation experiments were carried out using high energy collision dissociation (HCD) at a collision energy of 25eV.

For quantitation of enzyme reaction products in order to determine V_max_ and K_m_ values a unique sample preparation was employed. Dried enzyme assay samples were reconstituted in 200 μL of 10 mM ammonium acetate containing 1 μM [^13^C_10_^15^N_5_] adenosine, [^15^N_5_] adenine, [^15^N_4_] inosine (all obtained through Cambridge Isotope laboratories) and [^13^C_5_^15^N_2_] hypoxanthine (Santa Cruz Biotechnology Ltd., Dallas, TX, USA), thoroughly vortexed, sonicated and transferred to 300 μL glass vials and capped for analysis. Enzyme assay samples using substrate concentrations above 10 μM were further diluted to an effective concentration of 10 μM in the same reconstitution buffer to avoid concentrations above the linear analytical range of the instrumentation. Samples were run using the C18-PFP method described above on the Q Exactive Plus Orbitrap (using established instrumental parameters) with an injection volume of 2 μL. Blank injections were placed in between each test sample to eliminate carry-over effects.

#### LC-MS data processing

Data were acquired, processed and integrated using Xcalibur (Version 3.0, Thermo Fisher Scientific) and Compound Discoverer (Version 2.1, Thermo Fisher Scientific). For targeted analysis, metabolites of interest were identified using high resolution *m/z* values as specified in the METLIN database (Scripps Research Institute) corresponding to their [M+H]^+^ or [M-H]^–^ ion adducts in positive or negative ionisation modes, respectively. Compound retention time and fragmentation pattern were validated against known external standards. Peak areas corresponding to metabolite levels were manually quantified and normalized to internal standard or total ion content (as appropriate). NAD^+^/NADH ratios were quantified as relative levels and hence relative ratios have been provided.

For untargeted multivariate analysis performed as part of the screen for enzymatic activity and macrophage metabolomics, data were processed using Compound Discoverer (Version 2.1, Thermo Fisher Scientific) to determine unique LC-MS features with differential abundance between sample groups. For each differential MS feature, chromatogram peaks were manually verified using Xcalibur (Version 3.0, Thermo Fisher Scientific). Accurate *m/z* values of putative compounds were compared against the METLIN database (Scripps Research Institute) including [M+H]^+^, [M+Na]^+^, [M+NH_4_]^+^ for positive mode and [M-H]^–^, [M+Cl]^–^ for negative mode ion adducts with a mass tolerance of 2ppm. A combination of MS/MS fragmentation profile, molecular formulae calculation based on isotope pattern and expected chromatographic chemical behavior was then used to attribute metabolite identity. In case of ambiguity, and for all proposed FAMIN products and substrates, external standards were used to confirm metabolite identification. Data from positive and negative ionisation modes were combined and duplicate metabolite identities removed. Data was normalized to total ion content and fold change graphically depicted as volcano plots. Metabolite levels between groups were compared using a two-tailed, unpaired Student’s t test. For macrophage metabolomics Benjamini-Hochberg correction for multiple testing was applied.

For tracing experiments with [^13^C_10_^15^N_5_] adenosine, [^15^N_5_] adenine, [^13^C_1_^15^N_2_] guanosine, [^13^C_16_] palmitate, [^13^C_6_] glucose, [^13^C_5_^15^N_2_] glutamine, [^15^N_2_] glutamine, [^13^C_4_] fumarate and [^13^C_2_] fumarate, ^13^C and/or ^15^N incorporation into compounds were identified using accurate mass shift of +1.0034 and +0.9970, respectively. [^13^C_2_] fumarate contained ^13^C atoms at position 2 and 3. Unless otherwise indicated, cells were also control-pulsed with unlabelled reagent to determine effects of natural ^13^C abundance (1.1%) and ^15^N abundance (0.4%), with levels subtracted from quantified stable isotope incorporation as required. Mass isotopomer (MI) fraction was defined as the peak area of the MI divided by the total peak areas for all MIs expressed as a percentage.

#### Enzyme assays

Putative enzymatic function of recombinant human FAMIN (FAMIN^254I^) was investigated against an aqueous HepG2 metabolite library or nucleoside substrates at indicated concentrations and detected using UHPLC-MS as described above. The library consisted of the dried, Folch extracted aqueous phase from 5 × 10^6^ cells HepG2 cells 48h after transfection with *FAMIN* siRNA. Unless otherwise indicated in the figure legends, HEK293T cell-expressed protein was used for all assays. The reaction mixture (final volume 100 μL) consisted of 10 μg of recombinant protein and 10 μM nucleoside substrate in Dulbecco’s PBS (Thermo Fisher), pH 7.4 unless otherwise indicated. For the reverse reaction enzyme assay, 50 μM of adenine and ribose-1-phosphate were used. Protein elution buffer as described above was used as a control. The samples were incubated at 37°C for 1 h and then quenched with × 5 volume of ice-cold methanol. Samples were centrifuged at 21,000 *g* for 5 min transferred to fresh tubes and then dried down prior to analysis as described above.

#### Laccase assay

Laccase (benzenediol: oxygen oxidoreductase; EC 1.10.3.2) enzymatic activities of recombinant FAMIN^254I^, YfiH and YlmD were tested against the non-phenolic synthetic substrate ABTS [2,2-azinobis-(3-ethylbenzothiazoline-6-sulfonic acid)] as described (Gorshkov et al., 2017). 10 μg/mL of laccase from *Trametes versicolor* (positive control) or an equivalent quantity of purified FAMIN^254I^, YfiH or YlmD was used in all assays. The reaction mixture (final volume 100 μL) consisted of 1 mM ABTS, 0.1 mM CuSO_4_ and purified protein in Dulbecco’s PBS (Thermo Fisher), pH 7.4. The samples were incubated at 37°C and activity determined by measuring absorbance change at 405 nM. For LC-MS based laccase assays, the reaction mixture (final volume 100 μL) consisted of 10 μg of recombinant protein and 10 μM laccase substrate, sinapic acid or ferulic acid, in Dulbecco’s PBS (Thermo Fisher), pH 7.4 unless otherwise indicated. The samples were incubated at 37°C for 1 h and then quenched with × 5 volume of ice-cold methanol. Samples were centrifuged at 21,000 *g* for 5 min, transferred to fresh tubes and then dried down prior to analysis as described above.

#### Intracellular pH assay

Intracellular pH (pH_c_) was determined using the fluorogenic cytoplasmic pH indicator probe pHrodo Red AM (Thermo Fisher). M0, M1-polarized BMDMs, and HepG2 cells were incubated at 37°C for 30 min in Hank’s Balanced Salt Solution (HBSS) supplemented with 20mM HEPES and with pHrodo diluted at 5 μM final concentration. Cells were then washed with HEPES-HBSS and fluorescence measured using a microplate reader (Tecan infinite M1000) with an excitation/emission at 560/585nm. To assess the effects of exogenously supplied fumarate and malate on pH_c,_ M0 and M1-polarized BMDMs were incubated 24 h prior to pHrodo assay with medium supplied with 300 μM fumarate, 300 μM malate or vehicle control. To evaluate the effects of *L*-alanosine treatment on pH_c_, HepG2 cells and M1 macrophages were treated 24 h prior to pHrodo assay with 100uM *L*-alanosine or at the concentration indicated in figure legends in the presence or absence of 300 μM fumarate, 300 μM malate or vehicle control. pH_c_ was alternatively assessed in M1-polarized macrophages using a dual-excitation ratiometric pH indicator, BCECF AM probe (Thermo Fisher). In brief, cells were loaded with 5 μM of BCECF diluted in HBSS and incubated in a non-CO_2_ incubator at 37°C for 30 min. Cells were washed twice with HBSS and signals measured using a microplate reader (Tecan infinite M1000) with dual excitation set at 490 nm and 440 nm and fixed emission at 535 nm.

#### Oxygen consumption rate and extracellular acidification rate

HepG2 cells were directly plated (7 × 10^3^ cells/well) and transfected with specific targeting siRNAs (Dharmacon) for 48 h in XF-96 cell culture plates. BMDMs (7 × 10^4^ cells/well) were plated in XF-96 plates and differentiated as described above toward M1 macrophages or left unpolarized and assayed as M0 macrophages. For both BMDMs and HepG2, cells were then washed and incubated for 1 h in XF assay medium (unbuffered DMEM pH 7.4 with 10 mM glucose and 2 mM L-glutamine) in a non-CO_2_ incubator at 37°C as per manufacturer’s instructions (Seahorse Agilent). Real time measurements of extracellular acidification rate (ECAR) and oxygen consumption rate (OCR) were performed using an XF-96 Extracellular Flux Analyzer (Agilent). Three or more consecutive measurements were obtained under basal conditions and after the sequential addition of 1 μM oligomycin, to inhibit mitochondrial ATP synthase; 1.5 μM FCCP (fluoro-carbonyl cyanide phenylhydrazone), a protonophore that uncouples ATP synthesis from oxygen consumption by the electron-transport chain; and 100 nM rotenone plus 1 μM antimycin A, which inhibits the electron transport chain. To assess glycolysis, three or more consecutive ECAR measurements were obtained under basal conditions and after the sequential addition of 1 μM oligomycin, to elicit maximal glycolytic capacity and 100 mM 2-DG (2-deoxyglucose) to inhibit glycolysis. To assess the effects of *L*-alanosine treatment on OCR and ECAR, cells were exposed to 60 μM (HepG2 cells) or 100 μM (BMDMs) of *L*-alanosine for 24 h or as indicated in the figure legends prior to Seahorse assay. To assess the effects of SB204990 on OCR and ECAR, M0 macrophages were treated with 5 μM of SB204990 for 3 h followed by Seahorse assay. To evaluate the effects of exogenously supplied fumarate or malate on OCR and ECAR, cells were treated 24 h with 250 μM fumarate or malate (HepG2 cells) or 8 h with 300 μM (M0 macrophages) prior to Seahorse assay. OCR was also assessed via Seahorse assay in *Famin*^p.254I^ M0 macrophages silenced for *Adss*, *Adsl or for Ampd*s *(Ampd1, Ampd2 and Ampd3).* Gene silencing in those cells was achieved using an electroporation-based transfection method (Amaxa Nucleofection), according to manufacturer’s protocol (Lonza). siRNA of interest was purchased from Dharmacon (Horizon Discovery) and used for nucleofection at final concentration of 300nM.

#### Transfection of siRNA into HepG2 cells

Gene silencing in HepG2 cells was achieved through lipofection using the Lipofectamine RNAiMAX transfection reagent (Thermo Fisher) coupled to an optimized “reverse transfection” protocol for that cell line as per RNAiMAX manufacturer’s protocol (https://www.thermofisher.com/uk/en/home/references/protocols/cell-culture/transfection-protocol/rnaimax-reverse-transfections-lipofectamine.html). For all experiments using siRNA transfection into HepG2 cells, the targeting siRNA of interest was purchased from Dharmacon (Horizon Discovery) and used at final concentration of 25nM.

#### RNA extraction and sequencing

RNA was extracted using RNeasy Mini Kit (QIAGEN, 74104) with on-column DNase digestion, in accordance with the manufacturer’s instructions, and RNA quantified with a NanoDrop ND-1000 spectrophotometer. RNA quality was assessed using the Agilent 2200 TapeStation system. For all samples RNA integrity number was 9.7 or above. Library preparation was performed using the TruSeq stranded mRNA library prep kit (Illumina, 20020594), with 400ng total RNA per sample in accordance with the manufacturer’s instructions. Eleven libraries were sequenced at a time in one lane on an Illumina HiSeq3000 (1x50 base pairs). FastQ files were quality-checked (FastQC; http://www.bioinformatics.babraham.ac.uk/projects/fastqc/) and any residual adaptor sequences were removed (TrimGalore; http://www.bioinformatics.babraham.ac.uk/projects/trim_galore/). Reads were then aligned to the reference genome (mm10, UCSC) using HISAT2 (Kim et al., 2015). Differential gene expression analysis was performed on read count files using the limma package in R with the Voom transformation. Gene Set Enrichment Analysis was performed using log counts-per-million (CPM) following batch correction (sva package in R).

#### Phylogenetic analysis of FAMIN orthologs

The protein sequences were concatenated and aligned with MAFFT v. 7.20 (RRID: SCR_011811) and maximum-likelihood trees were constructed using RAxML v. 8.2.8 (RRID: SCR_006086) with the standard LG model and 100 rapid bootstrap replicates. Trees were visualized using FastTree followed by iTOL.

#### Immunoblot

Immunoblot analysis was performed as per standard procedures. In brief, cells were washed with ice-cold PBS and lysed in RIPA buffer (50 mM Tris pH 7.4, 150 mM NaCl, 1% Igepal, 0.5% sodium deoxycholate, 0.1% SDS) supplemented with protease and phosphatase inhibitors. Protein concentration was measured using a Pierce BCA protein assay kit and equal amounts of proteins loaded onto SDS polyacrylamide gels. Proteins were transferred onto nitrocellulose membrane using a Trans-Blot Turbo transfer system (Bio-Rad). Membranes were incubated for 1 h at room temperature in blocking buffer (5% skimmed milk), followed by incubation overnight with primary antibody. Antibodies were detected using HRP-conjugated secondary antibody and visualized using 20 × LumiGLO Reagent (Cell Signaling).

#### Mitochondrial ROS

For mitochondrial ROS measurements, macrophages were seeded at 4.5 × 10^4^ cells per well in a 96-well half area microplate (Greiner) and polarized as described above. Macrophages were then washed with warm phenol red-free Dulbecco’s Phosphate Buffered Saline (DPBS), with CaCl_2_ and MgCl_2_. Cells were incubated for 30 min at 37°C with 5 μM of the mitochondrial superoxide indicator probe, MitoSox (Invitrogen), diluted in the same wash medium. Fluorescence intensity was measured after washing using a plate reader (TECAN Infinite M1000) with excitation/emission at 510/580nm. To evaluate the effects of exogenously supplied fumarate on mitochondrial ROS, M1 macrophages were treated with 300 μM fumarate for 24 h prior to the assay.

#### Mitochondrial membrane potential and biomass

Mitochondrial membrane potential was assessed using the TMRE probe (abcam). Macrophages were seeded at 4.5 × 10^4^ cells per well in a 96-well half area microplate (Greiner) and polarized toward M1 as described above. Cells were incubated for 30 min with TMRE at 100 nM diluted in warm culture medium (RPMI). Cells were then washed twice with warm PBS / 0.2% BSA and fluorescence intensity detected using a plate reader with excitation/emission at 549/575 nm. As indicated, cells were incubated with 5 μM FCCP for 15min prior to adding TMRE. For determination of mitochondrial biomass, M1-polarized microphages were incubated for 30 min with 100 nM of MitoTracker Green (Thermo Fisher) diluted in warm phenol-free, serum-free RPMI medium. Fluorescence intensity was measured using a plate reader (TECAN Infinite M1000) with excitation/emission at 490/516 nm.

#### Cell proliferation assay

Proliferation rate of HepG2 cells was determined using the CyQUANT Proliferation Assay (Thermo Fisher) following the manufacturer’s protocol. In brief, cells were transfected and cultured in black polystyrene microplates with a flat clear bottom (Greiner CELLSTAR). At the indicated time points, fluorescence intensity was measured using a plate reader (Infinite M1000 Pro Multi Detection, TECAN) with appropriate wavelength.

### Quantification and Statistical Analysis

Statistical analyses were performed using Graphpad Prism 7 and 8 or Compound Discoverer (Thermo Scientific) as described in LC-MS analysis methods. Statistical parameters including the statistical test used, exact value of *n*, what *n* represents, and measures of distribution and deviation are reported in the figure legends. Unless otherwise stated, statistical significance was calculated as appropriate using unpaired, two-tailed Student’s t test or one-way ANOVA with a *P value* of < 0.05 considered significant and applicable post hoc testing. Images utilized within figure schematics have been adapted from https://smart.servier.com/. Data are represented as individual data points with the mean and standard error of the mean (SEM).

### Data and Code Availability

The data that support the findings of this study are available from the corresponding author upon reasonable request. RNA-Seq data generated in this paper can be accessed at the Gene Expression Omnibus (GEO: GSE126641). Crystallography data for Apo YlmD (PDB: 6T0Y) and inosine-bound YlmD (PDB: 6T1B) have been deposited at the worldwide Protein Data Bank.

## Supplemental Information

Table S1. FAMIN Variation Linked to Human Disease, Related to Figure 1

Table S2. Recombinant FAMIN-Dependent Metabolism of ^13^C_10_^15^N_5_-Adenosine, Related to Figure 2

Table S3. Crystallographic Data Collection and Refinement Statistics for YlmD from *Geobacillus stearothermophilus*, Related to Figure 3

Table S4. FAMIN^254I^ Enzyme Kinetics, Related to Figure 3

Table S5. Metabolite Levels in FAMIN-Overexpressing HEK293T Cells, Related to Figure 3

Table S6. ^13^C_10_^15^N_5_-Adenosine, ^15^N_5_-Adenine, ^13^C_1_^15^N_2_-Guanosine, ^13^C_16_-Palmitate, ^13^C_6_-Glucose, ^13^C_5_^15^N_2_-Glutamine, ^13^C_2_-Fumarate, and ^13^C_4_-Fumarate Labeling in *Famin*^p.254I^, *Famin*^p.254V^, and *Famin*^p.284R^ Macrophages, Related to Figures 4, 5, 6, and 7

Table S7. Differentially Expressed Genes between *Famin*^p.254I^ and *Famin*^p.284R^ M1 Macrophages, Related to Figure 5
