# FAMIN Is a Multifunctional Purine Enzyme Enabling the Purine Nucleotide Cycle

**DOI:** 10.1016/j.cell.2020.02.005

**Published:** 2020-02-20

**Authors:** M. Zaeem Cader, Rodrigo Pereira de Almeida Rodrigues, James A. West, Gavin W. Sewell, Muhammad N. Md-Ibrahim, Stephanie Reikine, Giuseppe Sirago, Lukas W. Unger, Ana Belén Iglesias-Romero, Katharina Ramshorn, Lea-Maxie Haag, Svetlana Saveljeva, Jana-Fabienne Ebel, Philip Rosenstiel, Nicole C. Kaneider, James C. Lee, Trevor D. Lawley, Allan Bradley, Gordon Dougan, Yorgo Modis, Julian L. Griffin, Arthur Kaser

(Cell *180*, 278–295.e1–e23, January 23, 2020)

Due to a production error, the last name of author Ana Belén Iglesias-Romero was misspelled as “Inglesias-Romero.” This error has been corrected online, and we apologize for any confusion we may have caused.

