# Supplementary material for: FAMIN Is a Multifunctional Purine Enzyme Enabling the Purine Nucleotide Cycle
Source: Cell. 2020 Jan 23;180(2):278–295.e23. doi: 10.1016/j.cell.2019.12.017 (PMC6978800; doi:10.1016/j.cell.2019.12.017)
Supplement: Table S1. FAMIN Variation Linked to Human Disease, Related to Figure 1 [file mmc1.pdf]

Cell, Volume *180*

Supplemental Information

FAMIN Is a Multifunctional Purine Enzyme

Enabling the Purine Nucleotide Cycle

M. Zaeem Cader, Rodrigo Pereira de Almeida Rodrigues, James A. West, Gavin W. Sewell, Muhammad N. Md-Ibrahim, Stephanie Reikine, Giuseppe Sirago, Lukas W. Unger, Ana Belén Inglesias-Romero, Katharina Ramshorn, Lea-Maxie Haag, Svetlana Saveljeva, Jana-Fabienne Ebel, Philip Rosenstiel, Nicole C. Kaneider, James C. Lee, Trevor D. Lawley, Allan Bradley, Gordon Dougan, Yorgo Modis, Julian L. Griffin, and Arthur Kaser

Supplemental Table 1. FAMIN variation linked to human disease

Rare variants – monogenic disorders	Clinical presentation	Laboratory findings	Classification	Age of onset	Background	Ref
c.T850C p.C284R autosomal recessive	Quotidian fever, erythematous maculopapular rash; symmetrical polyarthritis of small and large joints; 9/13 with persistent systemic manifestation with intermittent fever and active polyarthritis; 4/13 with short-lived systemic manifestation and polyarticular subtype	Leukocytosis, thrombocytosis, high inflammatory markers; 8/13 ANA; 5/13 weak RF	**sJIA** (Still's disease)	3.2±1.8 yrs	13 patients in 5 consanguineous Saudi Arabian families	(Wakil et al., 2015)
c.T850C p.C284R autosomal recessive	Early-onset Crohn's disease; severe debilitating arthropathy		**Early-onset inflammatory bowel disease**	< 10 yrs	4 patients in consanguineous Saudi Arabian family	(Patel et al., 2014)
c.128_129delGT p.C43Yfs*6 autosomal recessive	RF-negative polyarticular juvenile idiopathic arthritis: chronic, symmetric polyarthritis of large and small joints, erosive in 2/3. No fever, skin rash, uveitis, or other extra-articular manifestations.	Leukocytosis, thrombocytosis, anaemia, high inflammatory markers	**JIA**	2-4 yrs	3 patients in consanguineous Moroccon family	(Arostegui et al., 2015)
c.827delC p.T276fs*2 autosomal recessive	Child 1: at 16 months severe polyarthritis of small & large joints, lymphadenopathy, quotidian fever without serositis or exanthema. Precursor B cell acute lymphoblastic leukaemia at age 6. Child 2: large joint arthritis, later systemic polyarthritis	Leukocytosis, high inflammatory markers	(1) **sJIA** (Still's disease) (2) extended oligoarticular **JIA**	15 and 16 months	2 siblings in consanguineous Lebanese family	(Kallinich et al., 2016)
c.3G>A p.0 autosomal recessive	Polyarticular arthritis; 1/4 with fever or rash; 3/4 chronic, 1/4 episodic; 1/4 growth retardation		Polyarticular **JIA**	<1 yr (3/4); 2 yr (1/4)	4 patients in consanguineous family	(Karacan et al., 2018)
c.1240C>T p.R414X autosomal recessive	Chronic polyarticular arthritis; 1/3 fever or rash; 2/3 splenomegaly		Polyarticular **JIA**	<1 yr (2/3); 2.5 yr (1/3)	3 patients in consanguineous family	(Karacan et al., 2018)
c.988_990del p.I330del autosomal recessive	Fever, rash, pericarditis; chronic polyarticular arthritis; growth retardation		**sJIA** (Still's disease)	<1yr and 2 yrs	2 patients in consanguineous family	(Karacan et al., 2018)
Compound: Het: c.1109G>A p.C370Y; Hom: c.760A>G [rs3764147] p.I254V	1/2 episodic enthesitis-related juvenile arthritis; 1/2 episodic oligoarticular juvenile arthritis; 1 unaffected sibling with identical genotype		**JIA**	10 and 7 yrs	2 patients in consanguineous family	(Karacan et al., 2018)
**Common variants**	**Minor allele frequency**	**Odds ratio**	**Disease risk**		**Population**	**Ref**
rs3764147 c.760A>G p.I254V	~0.26	1.25	**Crohn's disease**		Caucasian	(Barrett et al., 2008)
rs3764147 c.760A>G p.I254V	~0.35	1.68	**Leprosy**		Han Chinese	(Zhang et al., 2009)
rs2121033 (intronic) (rs9316059, intronic)	~0.28	0.69 – 0.79	**Behçet's disease**		Turkish, Iranian and Japanese	(Takeuchi et al., 2017)

p.I254V: reduced ROS production, oxygen consumption (oxidative phosphorylation) and extracellular acidification (glycolysis) (Cader et al., 2016)

p.C284R: loss-of-function, phenotype indistinguishable from knock-out allele (Cader et al., 2016)

Abbreviations: ANA, anti-nuclear antibody; JIA, juvenile idiopathic arthritis; RF, rheumatoid factor; ROS, reactive oxygen species; sJIA, systemic juvenile idiopathic arthritis
